# Transcriptional responses of brain endothelium to *Plasmodium falciparum* patient-derived isolates *in vitro*

**DOI:** 10.1128/spectrum.00727-24

**Published:** 2024-06-12

**Authors:** Caroline Askonas, Janet Storm, Grazia Camarda, Alister Craig, Arnab Pain

**Affiliations:** 1 Pathogen Genomics Laboratory, Bioscience Program, Biological and Environmental Sciences and Engineering (BESE) Division, King Abdullah University of Science and Technology, Thuwal, Saudi Arabia; 2 Tropical Disease Biology, Liverpool School of Tropical Medicine, Liverpool, United Kingdom; Weill Cornell Medicine, New York, New York, USA

**Keywords:** cerebral malaria, cytoadherence, *Plasmodium falciparum*, HBMEC, endothelium, gene expression

## Abstract

A hallmark of cerebral malaria (CM) is sequestration of *Plasmodium falciparum*-infected erythrocytes (IE) within the brain microvasculature. Binding of IE to endothelium reduces microvascular flow and, combined with an inflammatory response, perturbs endothelial barrier function, resulting in breakdown of the blood-brain barrier (BBB). Cytoadherence leads to activation of the endothelium and alters a range of cell processes affecting signaling pathways, receptor expression, coagulation, and disruption of BBB integrity. Here, we investigated whether CM-derived parasites elicit differential effects on human brain microvascular endothelial cells (HBMECs), as compared to uncomplicated malaria (UM)-derived parasites. Patient-derived IE from UM and CM clinical cases, as well as non-binding skeleton-binding protein 1 knockout parasites, were overlaid onto tumour necrosis factor (TNF)-activated HBMECs. Gene expression analysis of endothelial responses was performed using probe-based assays of a panel of genes involved in inflammation, apoptosis, endothelial barrier function, and prostacyclin synthesis pathway. We observed a significant effect on endothelial transcriptional responses in the presence of IE, yet there was no significant correlation between HBMEC responses and type of clinical syndrome (UM or CM). Furthermore, there was no correlation between HBMEC gene expression and both binding itself and level of IE binding to HBMECs, as we detected the same change in endothelial responses when employing both binding and non-binding parasites. Our results suggest that interaction of IE with endothelial cells in this co-culture model induces some endothelial responses that are independent of clinical origin and independent of the expression of the major variant antigen *Plasmodium falciparum* erythrocyte membrane protein 1 on the IE surface.

**IMPORTANCE:**

Cerebral malaria (CM) is the most prevalent and deadly complication of severe *Plasmodium falciparum* infection. A hallmark of this disease is sequestration of *P. falciparum*-infected erythrocytes (IE) in brain microvasculature that ultimately results in breakdown of the blood-brain barrier. Here, we compared the effect of *P. falciparum* parasites derived from uncomplicated malaria (UM) and CM cases on the relative gene expression of human brain microvascular endothelial cells (HBMECs) for a panel of genes. We observed a significant effect on the endothelial transcriptional response in the presence of IE, yet there is no significant correlation between HBMEC responses and the type of clinical syndrome (UM or CM). Furthermore, there was no correlation between HBMEC gene expression and both binding itself and the level of IE binding to HBMECs. Our results suggest that interaction of IE with endothelial cells induces endothelial responses that are independent of clinical origin and not entirely driven by surface *Plasmodium falciparum* erythrocyte membrane protein 1 expression.

## INTRODUCTION

In spite of global efforts to reduce mortality and morbidity of malaria, an estimated 249 million cases and 608,000 deaths were reported in 2022, with the majority occurring in children under the age of 5 (1). The majority of symptomatic infections results in mild febrile cases of uncomplicated malaria (UM), as severe disease only occurs in 1%–2% of all cases (2). Out of the eight *Plasmodium* spp. that have been reported to cause either direct or zoonotic infection in humans, *Plasmodium falciparum* is considered a major driver of severe disease and mortality (3, 4). Cerebral malaria (CM) is a severe neurological complication of malaria infection that is a major cause of death, with mortality estimated between 15% and 25% and clinical manifestations including unarousable coma and seizures (5, 6). Mortality in pediatric CM is associated with brain swelling, which is considered to occur as a result of increased permeability of the blood-brain barrier (BBB) due to loss of BBB function, leading to vasogenic edema (6, 7). Continued study of the pathophysiology of CM is required to develop targeted treatment therapies.

It is evident that CM is a complex, multi-component disease requiring a multifactorial view of pathogenesis that considers the heterogeneous clinical manifestations of CM (8, 9). Sequestration of *P. falciparum*-infected erythrocytes (IE) to brain microvasculature is a hallmark of CM. Cytoadhesion of IE to the endothelium is mediated by *Plasmodium falciparum* erythrocyte membrane protein 1 (*Pf*EMP1), a variable surface protein that is expressed by IE and contains an extracellular binding region (10). This polymorphic protein is encoded by a group of approximately 60 *var* genes with monoallelic expression, and the composition of the extracellular region confers binding to various host receptors (11). Several vascular surface proteins, in particular CD36, EPCR, and ICAM-1, may serve as IE adhesion receptors, and expression of these endothelial receptors varies between vascular locations and is modulated in part by cytokines, such as TNF and interferon-ɣ (IFN-ɣ) (9, 10, 12, 13). EPCR is associated with severe malaria, as *Pf*EMP1s containing EPCR binding domains have been linked to brain swelling in pediatric CM (14–17). While ICAM-1 mediates cytoadhesion related to sequestration in the brain (13–15), its direct role in the pathology of CM is less clear. However, research has demonstrated that a subset of EPCR-binding parasites has the ability to bind ICAM-1 and that expression of these dual binders has been associated with CM in patients (15, 18, 19). Cytoadherence alters a range of endothelial cell processes and leads to the activation of endothelium as well as reducing microvascular flow. Accumulation of IE causes vessel occlusion and generates microenvironments where both parasite factors and soluble erythrocytic content accumulate after IE rupture (8, 20). Ultimately, the contributions of microvascular obstruction and endothelial dysfunction lead to the disruption and breakdown of the BBB (5, 8, 9, 17).

Although IE derived from malaria patients have exhibited different binding phenotypes, most studies employ well-characterized laboratory strains to investigate cytoadherence and interaction of different *Pf*EMP1 variants to brain endothelial cells (19, 21–24). Separate investigations have highlighted the divergent effect of both TNF stimulation and IE cytoadhesion on endothelial transcriptional responses and the pathways they induce (23, 25). IE isolated from peripheral blood of UM and CM patients have been used to study cytoadherence to human brain microvascular endothelial cells (HBMECs). It was found that IE isolated from CM patients exhibit differential binding capacities to TNF-activated brain endothelium *in vitro*, as they were associated with increased adhesion (26). Further study employing patient-derived isolates is needed to elucidate the impact of parasite cytoadhesion on the brain microvasculature in association with different disease outcomes.

The aim of this study is to investigate whether CM-derived IE elicit differential effects on HBMECs as compared to UM-derived isolates. An *in vitro* co-culture model of cytoadherence was employed wherein patient-derived IE isolates were overlaid onto TNF-activated HBMECs and the relative gene expression of HBMECs after incubation with either red blood cells (RBC) or IE for a panel of genes was assessed. A study utilizing two *P. falciparum* lab strains with different binding capacities to HBMECs found that different *Pf*EMP1-expressing variants induced divergent endothelial transcriptional responses during cytoadherence (25). Differentially expressed genes (DEGs) were identified in HBMECs in pathways involved in the pathology of severe malaria, such as inflammation, apoptosis, and barrier integrity. From this list of DEGs, a panel of genes was compiled, and gene expression was investigated by qPCR using the Fluidigm DELTAgene Assays system. First, the relative gene expression of HBMECs after exposure to control RBC or IE for combined UM- and CM-derived isolates was compared, followed by comparing the expression data between clinical types. We observed variation in endothelial transcriptional responses to individual patient-derived IE as compared to RBC, but these responses did not show a significant differential effect between the UM- or CM-derived isolates. It was assessed whether the HBMEC relative gene expression after co-culture was correlated with the binding avidity of the parasite isolates, but a significant relationship was not observed. Our data confirm that IE have a profound effect on endothelial transcription that is specific to the presence of the *P. falciparum* parasites in the erythrocyte. However, we did not observe effects that were associated with disease severity with the panel of genes used in this study.

## MATERIALS AND METHODS

### Culture of endothelial cells

Primary HBMECs were obtained from Cell Systems, USA (ACBRI 376) and cultured in Endothelial Cell Growth Medium 2 (EGM2; C-22111, Promocell) supplemented with 2% fetal calf serum, 5 ng/mL epidermal growth factor, 10 ng/mL basic fibroblast growth factor, 20 ng/mL Long R3 IGF-1, 0.5 ng/mL vascular endothelial growth factor (VEGF) 165, 1 µg/mL ascorbic acid, 22.3 µg/mL heparin, and 0.2 µg/mL hydrocortisone from the supplement pack (C-39211, Promocell) in a 5% CO_2_ incubator at 37°C. For endothelial cells to adhere to the surface, flasks were coated with attachment factor (10308363, Gibco). Cells were passaged using Accutase solution (A6964, Sigma Aldrich), following manufacturer instructions. The receptor expression of HBMECs was characterized by flow cytometry as described by Storm et al. (26).

### Culture of *P. falciparum* parasites

Patient-derived *P. falciparum* parasites were isolated from peripheral blood from pediatric malaria cases recruited at the Queen Elizabeth Central Hospital, Blantyre, Malawi, with ethical approval from the College of Medicine, University of Malawi and LSTM, as described in Storm et al. (26). Four UM and four CM patient-derived isolates were selected based on their differential cytoadherence profile to HBMECs and human dermal microvascular endothelial cells and their ability to establish in continuous culture (Fig. 3). After isolation from blood, limited IE could be cryopreserved, especially for CM isolates, and only a maximum of 150 µL IE could be frozen. Establishing the isolates as a continuous culture, expanding the cultures to generate more stabilates, and subsequently have 65 mL of culture volume at 2% hematocrit (HCT) for the co-cultures require a considerable amount of time. Therefore, co-cultures were performed after at least 21 days in culture (DIC). The total DIC for the patient-derived parasite isolates for co-culture 1 and 2, respectively, were UM1: 24 and 43, UM2: 21 and 32, UM3: 31 and 42, UM4: 24 and 25, CM1: 29 and 29, CM2: 35 and 44, CM3: 49 and 59, and CM4: 29 and 41. The skeleton-binding protein knockout (SBP1-KO) lab strain was obtained from A.G. Maier and cultured in the presence of 4 nM WR99210 (27).

The IE were cultivated at 2% hematocrit in O+ human erythrocytes and grown at 37°C in multiple 25 cm^2^ tissue culture flasks (T25) filled with a gas mixture of 96% nitrogen, 3% carbon dioxide, and 1% oxygen. The duration of parasite culturing was isolate dependent, as different lengths of continuous culture were required to generate enough material to perform the experiments, ranging from 21 to 60 DIC. IE were maintained in complete RPMI 1640 medium with 11 mM glucose and 0.2% sodium bicarbonate (R-0883, Sigma) supplemented with 5% human serum, 0.25% Albumax II (11021029, ThermoFisher Scientific), 30 mM of HEPES (15630122, Gibco), 2 mM L-glutamine (G-7513, Sigma), 25 ng/mL gentamicin sulfate solution (G-1271, Sigma), and 0.11 mM hypoxanthine (H9636, Sigma), pH 7.4. Parasite growth was monitored using Giemsa staining (1092040500, Merck), and IE were passaged to maintain 2%–5% parasitemia. Mixed-staged cultures were synchronized for ring stages using a 5% D-sorbitol solution (S3889, Sigma), and IE at trophozoite stage were used for the *in vitro* co-culture studies with HBMECs. EC cultures, parasite lines, culture media, and washed RBCs were monitored for mycoplasma contamination using MycoBlue Mycoplasma Detector (Vazyme, D101-02).

### Co-culture of HBMECs and IE

HBMECs at passages 5–8 were cultured until 90% confluency in T25 flasks. One day prior to co-culture, HBMECs were seeded in 12-well plates at a density of 50,000 cells/cm^2^ (for 3.8 cm^2^ wells, this is equivalent to total of 190,000 cells) in the morning and stimulated overnight with 10 ng/mL TNF (PHC3015, Invitrogen). The RBC control was prepared by culturing a 2% hematocrit suspension in RPMI medium overnight. On the day of the co-culture, the supernatant was removed from the HBMECs and replaced with EGM2 without heparin and hydrocortisone (EGM2min) 2 hours prior to co-culture. IE were enriched for mature stages by layering onto a 0.7% gelatin solution and incubation at 37°C for 45–60 minutes, and after enrichment, the hematocrit and parasitemia were calculated and IE suspensions were prepared to an average parasitemia of 30% (parasitemia ranged from 20% to 40%, depending on enrichment properties of the isolates) at 1% hematocrit in EGM2min.

The RBC control was also subjected to gelatin treatment, and a suspension at 1% hematocrit in EGM2min was prepared. EGM2min was used to maintain healthy HBMECs and did not affect parasite viability in the 6-hour co-culture, but parasite development seemed delayed, as assessed by microscopy. Medium was removed from the HBMECs, and 700 µL IE or RBC suspension was overlaid onto HBMECs for 6 hours in a 5% CO_2_ incubator at 37°C, as well as EGM2min as an additional control. Seven hundred microliters of 30% parasitemia at 1% hematocrit is equivalent to 2.1 × 10^7^ IE, and addition of the IE suspension to HBMECs is equivalent to a ratio of 110 IE/HBMECs. A 0-hour control with EGM2min was used to compare with the 6-hour time points. Control samples were generated by overnight incubation of HBMECs with either 10 ng/mL of TNF or 1 ng/mL IL-1β (579402, BioLegend) and compared to EGM2min, for which samples were collected directly after stimulation.

At each time point, the co-culture medium was removed, and the HBMECs were washed once with EGM2min to remove unbound IE or uninfected RBC. Cells were directly lysed in the well using 350 µL of lysing solution (1% 2-mercaptoethanol in Buffer RLT; RNeasy Mini Kit, 74106, Qiagen) by pipetting over the well. The lysates were collected into a 1.5 mL sterile tube, vortexed, and stored at −80°C until RNA extraction. Two independent co-culture experiments were performed for each patient isolate and SBP1-KO.

In parallel to the co-culture experiments, activation of HBMECs after overnight incubation with TNF was assessed. One day prior to co-culture, HBMECs were plated onto 24-well plates at a density of 50,000 cells/cm^2^. The HBMECs were incubated overnight with either media alone or media containing 10 ng/mL of TNF. Cells were detached with Accutase, collected in phosphate buffered saline (PBS) + 1% fetal bovine serum (FBS) and stained for ICAM-1 expression on the endothelial cell surface using APC anti-human CD54 antibody (353111, BioLegend). Flow cytometry analysis was performed and confirmed that ICAM-1 expression was upregulated following overnight stimulation with TNF in each of the co-culture experiments.

### RNA extraction and quality assessment

Frozen HBMEC lysates were placed in a 37°C water bath until thawed and salts were dissolved. Total RNA was purified using the RNeasy Mini Kit (74106, Qiagen) following the manufacturer’s instructions using steps 4–10 of the “Purification of Total RNA from Animal Cells using Spin Technology” protocol. RNA was eluted in 50 µL of RNase-free water.

The RNA concentration of each sample was quantified using a Qubit fluorometer and the RNA High Sensitivity assays (Q32852, Invitrogen), following manufacturer instructions. The quality of the RNA samples was assessed using an Agilent 2100 Bioanalyzer (RNA 6000 Nano Kit, 5067-1511, Agilent technologies) following manufacturer instructions. The RNA integrity number (RIN) for all samples was between 9.4 and 10, indicating high quality RNA.

### Gene expression assays using the Fluidigm system and calculations

Gene expression qPCR assays were conducted on the extracted RNA for a panel of 49 genes that include pathways pertaining to inflammation, apoptosis, endothelial barrier function, and prostacyclin synthesis (listed in Table S1). In short, cDNA was prepared with reverse transcription before pre-amplification (13 cycles) and clean-up of the reactions using exonuclease 1, following the manufacturer protocols (100-6472 B1, PN 100-5875 C1) and reagents (Fluidigm, 100-6297; Fluidigm, 100-5580; New England Biolabs, M0293S; TEKnova, 10221). Real-time PCR data were collected using the Biomark HD system with a 96.96 Integrated Fluidic Circuit (IFC) chip using Fluidigm Deltagene Assays on the pre-amplified cDNA. The manufacturing protocol was followed for use of the Biomark HD system (PN 100-9792 A1) using the reagents specified for preparing samples (Biorad, 172-5211; Fluidigm, 100-7609) and assays (Fluidigm 100–7611; TEKnova, 10221). Technical replicates were performed for each sample, and Ct values were obtained as the throughput of the real-time data (Supplemental data). The primers for the gene panel were proprietarily designed, prepared, and tested by Fluidigm.

Relative gene expression was calculated using the 2^-ΔΔCt^ method as previously described (28) using glyceraldehyde-3-phosphate dehydrogenase (GADPH) as the endogenous control (ΔCt) and the 6-hour EGM2min-only sample as the normalization controls (ΔΔCt). In this way, the exponential fold changes (FCs, 2^-ΔΔCt^) in the target genes are calculated as normalized to the GAPDH internal control and relative to the expression of the HBMEC-media only at the 6-hour time point. For each target gene, the gene expression of HBMECs incubated with EGM2min at 6 hours is equal to 1 (baseline). Therefore, an FC >1 indicates increased relative gene expression and an FC <1 indicates decreased relative gene expression for the experimental samples. FCs were calculated for each technical triplicate of each sample for each experiment. To compare relative gene expression of HBMECs after incubation with various groups (e.g., RBCs, UM-derived parasite isolates, CM-derived parasites isolates, or combined IE), the mean, standard error of the mean (SEM), and confidence intervals for each group were calculated from the technical triplicates of each sample for each independent experiment. Either two-tailed, unpaired *t*-tests or Welch’s *t*-test was then performed to compare the relative gene expression between various corresponding groups. Additionally, a paired analysis was performed wherein the HBMEC-IE relative gene expression for each parasite isolate was compared to the corresponding HBMEC-RBC relative gene expression, for both experiments. For this analysis, the target FCs were calculated as normalized to GAPDH and relative to the corresponding experimental 6-hour EGM2min, and the mean and SD were determined from the technical triplicates for each sample. Both independent experiments for all isolates were performed for a 6-hour timepoint.

### Binding of parasite isolates to HBMECs under flow conditions

The binding assay was performed using the Cellix microfluidics system as previously described (26, 29). Briefly, HBMECs or HDMECs were stimulated overnight with 10 ng/mL TNF, detached, and seeded in attachment factor-coated Vena8 biochips (Cellix). When cells formed a confluent monolayer, an IE suspension of 2% parasitemia and 5% hematocrit in binding buffer (RPMI 1640 with 25 mM HEPES, 11 mM glucose, 2 mM glutamine, pH 7.2) was flowed through for 5 minutes at 37°C at a shear stress of 0.4 dyne/cm^2^. After a washing step, bound IE were counted by microscopy in 15 areas throughout the biochip and the mean IE/mm^2^ EC cell surface calculated.

### Statistical analysis

Statistical analyses were performed on Prism 9 (version 9.5.1, GraphPad Software). The exact statistical tests, along with the levels of significance, are detailed in the figure legends.

## RESULTS

### Expression of intermediates involved in maintaining vascular barrier integrity is increased in HBMECs in response to patient-derived *P. falciparum* parasites

To investigate endothelial responses to the patient-derived parasite isolates, we compared the relative gene expression of TNF-activated HBMECs after a 6-hour exposure to either RBC or IE derived from patients with clinically defined UM or CM. Additional media-only samples were collected to be used as normalization controls in the relative gene expression calculations.

The relative gene expression of 49 target genes was analyzed by grouping HBMEC samples by clinical type of the patient isolates, either HBMEC-UM or HBMEC-CM, and comparing them to the corresponding HBMEC-RBC control. Gene expression was calculated for HBMEC-UM, HBMEC-CM, and corresponding HBMEC-RBC by determining the mean FC ± SEMs for each group relative to the corresponding 6-hour EGM2min controls in each of the two independent experiments. Welch’s *t*-tests were performed to compare the relative gene expression of the HBMEC-IEs to their corresponding HBMEC-RBC controls, and the mean FC ± SEMs are reported in Table S1.

Differential gene expression was observed in HBMECs in the presence of IE compared to the corresponding RBC control for a number of genes, and several were selected for further study due to their relatively increased expression (FC >1.7), as well as the biological processes they are involved in. These genes encode B-cell lymphoma 2-related protein A1 (*BCL2A1*), cytochrome P450 family 1 subfamily A member 1 (*CYP1A1*), Krüppel-like factor 2 (*KLF2*), Krüppel-like factor 4 (*KLF4*), prostacyclin synthase (*PTGIS*), and prostaglandin-endoperoxide synthase 2 (*PTGS2*).

**Fig 1:**
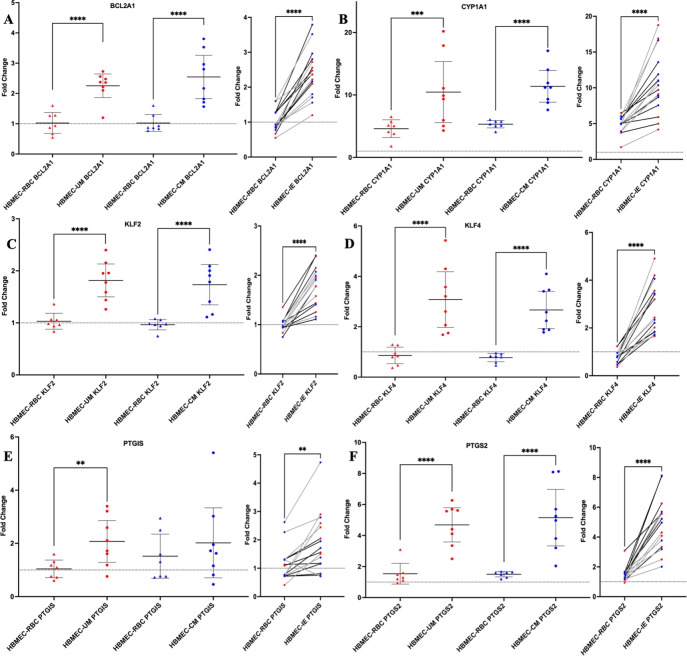
IE of both UM and CM clinical isolates affect HBMEC transcriptional responses as compared to RBC. Differential gene expression of HBMECs after 6-hour co-culture with patient-derived parasites compared to their corresponding RBCs for *BCL2A1* (A), *CYP1A1* (B), *KLF2* (C), *KLF4* (D), *PTGIS* (E), and *PTGS2* (F), calculated as relative to the corresponding 6-hour HBMEC-media control. Mean HBMEC gene expression (fold change) was compared in two ways for the selected genes: (left) Welch’s *t*-test comparing HBMEC-RBC and HBMEC-IE for UM or CM isolates and (right) paired *t*-test comparing all RBC controls and the combined HBMEC-IE of all isolates. The Welch’s *t*-test results shown in the graphs are from Fig. S1. Each point on the plot represents one sample FC, calculated as a mean of the technical triplicates, and the mean and 95% confidence intervals are indicated for each group. For the paired analysis, the mean FC was calculated from technical triplicates for each IE and corresponding RBC sample and are colored red for UM isolates and blue for CM isolates. A paired *t*-test was performed comparing the mean FC of HBMEC-IE with the corresponding HBMEC-RBC. Lines between the groups indicate that the RBC sample on the left was the control for the corresponding isolate sample on the right. Black lines represent samples from experiment 1, and gray lines represent samples from experiment 2. Two-tailed tests were performed for both analyses, and the dotted line in the plots marks the baseline of FC = 1. Significance is depicted by the *P*-value: *,0.01–0.05; **, 0.001–0.01; ***, 0.0001–0.001; ****, <0.0001.

A significant increase in expression of *BCL2A1*, *CYP1A1*, *KLF2*, *KLF4*, *PTGIS*, and *PTGS2* was observed for HBMECs after co-culture with both clinical isolate types as compared to the RBC controls (Fig. 1; Table S1). The mean HBMEC gene expression was compared in two ways: grouping by clinical syndrome compared to the corresponding RBC, and grouping by combining all IE samples compared to the corresponding RBC. The results for each gene are shown in Fig. 1, with a sub-figure showing the combined data for HBMEC-RBC and HBMEC-IE co-culture, with paired data points from the same experiment joined by a line. This allows visual verification of increased gene expression within each experiment, which corresponds to the increase in gene expression observed when samples are grouped by clinical type.

To validate the observed changes in gene expression, we assessed whether the Fluidigm panel would detect gene upregulation after incubating HBMECs overnight with known stimuli, IL-1β and TNF, and comparing the relative gene expression with a media control (Table S2). Indeed, incubation with these stimuli resulted in upregulation of HBMEC expression in the majority of tested genes, with large fold changes observed in both treatment groups for the following (IL-1β and TNF, respectively): *CXCL3* (1305 and 862), *ICAM1* (16 and 25), *PTGS2* (30 and 16), *SELE* (1305 and 862), and *VCAM1* (548 and 873).

Previous studies have demonstrated that TNF differentially regulates transcriptional effects on brain endothelial cells (23, 30), such as upregulated expression of *BCL2A1* in vascular endothelial cells (31); 0-hour data were collected to assess if the observed HBMEC FC expression was due to either activation with TNF or the effect of IE. To demonstrate the impact of TNF withdrawal, the relative gene expression of activated HBMECs after 2-hour (0-hour timepoint) and 8-hour (6 hour timepoint) incubation with media was compared for representative samples (Fig. S1). There was significant reduction of relative FC levels of *KLF4*, *PTGS2*, *ICAM-1*, *VCAM-1*, and an observed reduction in *BCL2A1* in HBMECs after withdrawal of TNF after 8 hours. These findings show that the differences in relative gene expression observed after co-culture are not artifacts of overnight TNF stimulation of the HBMECs.

### *P. falciparum* parasites derived from uncomplicated or cerebral malaria cases do not induce divergent HBMEC responses for the genes tested

**Fig 2:**
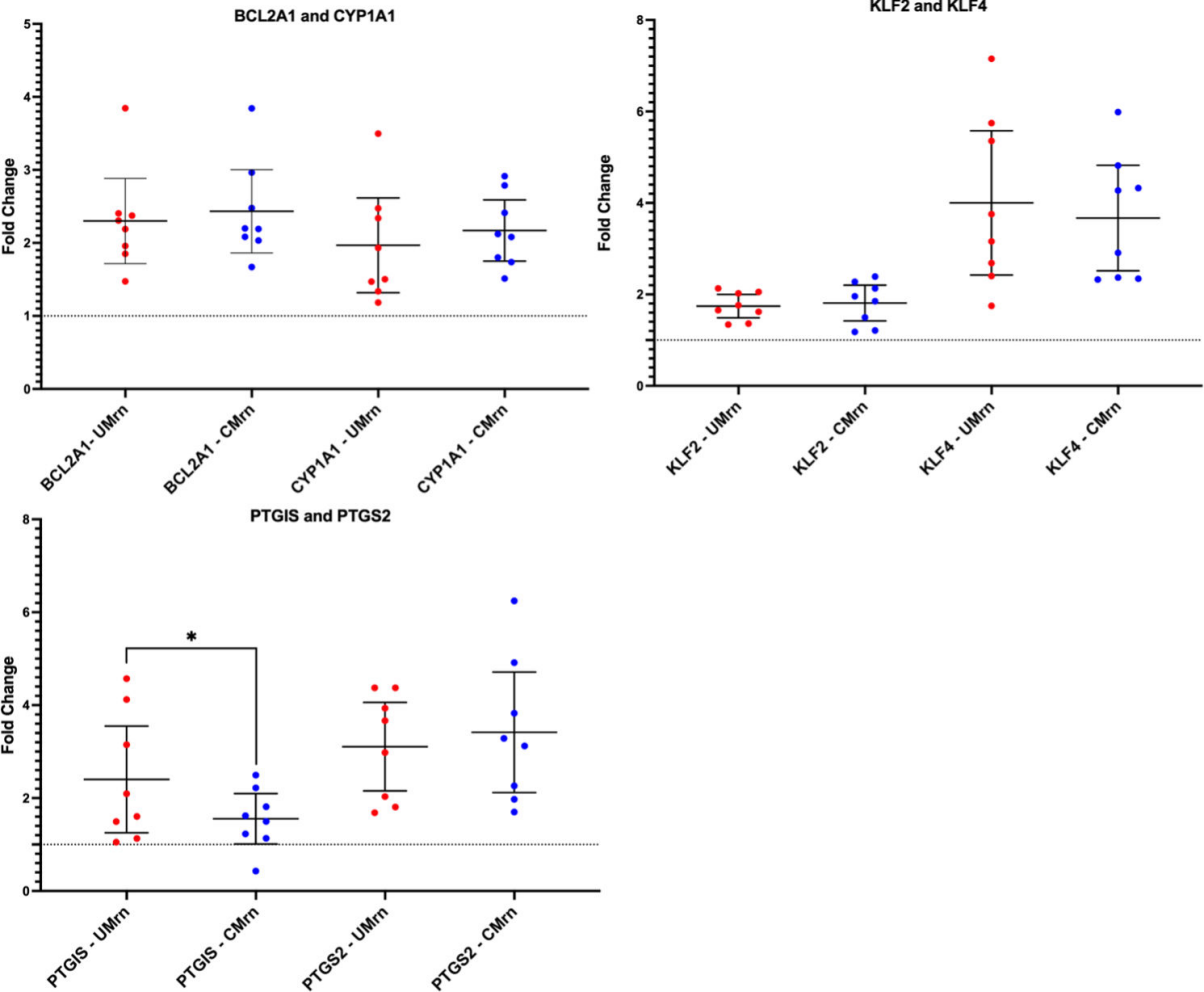
When normalized to HBMEC-RBC controls, isolates within clinical categories do not induce differential responses in HBMECs. To determine the effect of the parasites, HBMEC relative gene expression when exposed to UM and CM patient-derived isolates was calculated relative to HBMEC-RBC expression, using GAPDH as the endogenous control and the 6-hour HBMEC-RBC as the normalization sample reference. Each point represents one experimental sample (mean of triplicates), and the mean FC and 95% confidence intervals for each gene were calculated using both biological replicates (*n* = 8). RBC-normalized HBMEC-UM is represented as “UMrn,” and RBC-normalized HBMEC-CM is represented as “CMrn.” Significance between the two clinical categories was determined by two-tailed unpaired *t*-tests with *P*-value summary defined: *,0.01–0.05. The dotted line marks the baseline of FC = 1 (red, UM isolates; blue, CM isolates).

To determine whether the observed effect on HBMEC responses to the patient-derived IE was specific to the clinical syndrome, the effect of RBCs on HBMECs was normalized. To take into account variation in the RBCs for each experiment, gene expression FCs (2^-ΔΔCt^) were calculated relative to the corresponding 6-hour HBMEC-RBC sample, which was used as the normalization control (ΔΔCt). Mean gene FCs were calculated for each clinical isolate type (HBMEC-UM and HBMEC-CM) using the triplicates of all the experimental samples. Unpaired *t*-tests were performed to compare the mean relative gene expression of HBMEC-UM to HBMEC-CM (Fig. 2). Apart from *PTGIS*, there was no differential effect on HBMEC transcriptional responses when comparing UM- and CM-derived isolates.

**Fig 3:**
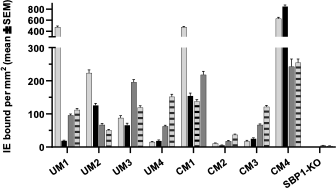
Binding of patient-derived isolates to HBMECs under flow conditions. Binding of IE to TNF-stimulated HBMECs or human dermal microvascular endothelial cells (HDMECs) was determined using microfluidics. Shown is the mean ± SEM IE/mm^2^ EC cell surface of 15 fields counted by microscopy. The level of binding for the UM- and CM-derived isolates shortly after isolation from the patients is depicted in light gray (HDMECs) and in black (HBMECs). After certain DIC binding of HBMECs is depicted in dark gray (experiment 2) and light gray with horizontal bars (experiment 1). UM1: 30 and 64 DIC, UM2: 32 and 50 DIC, UM3: 40 and 44 DIC, UM4: 25 and 38 DIC, CM1: 31 and 36 DIC, CM2: 32 and 46 DIC, CM3: 50 and 66 DIC, CM4: 42 and 46 DIC. SBP1-KO does not bind to HBMECs.

The isolates chosen for this study were from a panel of patient-derived *P. falciparum* parasites, isolated from pediatric uncomplicated and cerebral malaria cases in Malawi as described in Storm et al. (26). During extended periods of culture, and when establishing *in vitro P. falciparum* culture lines from patient parasite isolates, the *var* gene transcription profiles may change, which could affect phenotypic changes, such as binding to HBMECs (32, 33). To establish the isolates in culture and expand the volume to sufficient levels, the experiments were carried out after culturing the IE for 21–60 days. To determine if their adherence to HBMECs had changed due to *var* gene switching over time (32, 33), binding assays under flow conditions were performed as close to the length of culture time (DIC) for each co-culture experiment (Fig. 3). Compared to their initial binding phenotype, all isolates showed altered binding to HBMECs. UM1, UM3, UM4, CM2, and CM3 increased their binding after various days in culture, with some variation between the two sets of DIC, and UM2 and CM4 decreased their binding. The SBP1-KO strain does not bind to HBMECs.

Using data from both experiments, the binding capacities of the isolates were then correlated with the mean gene expression (FC calculated using the 6-hour RBC as the normalization control) of HBMECs following co-culture with IE using Spearman’s correlation. These calculations were performed for the entire gene panel by grouping the data per experiment, and representative plots for *KLF4*, *PTGIS*, and *PTGS2* are presented in Fig. S2A and B for both experiments. After co-culture, there was no observed significant correlation between the binding avidity of the parasites and the HBMEC gene expression.

### Observed differential effects of *P. falciparum* parasites on HBMEC responses are not solely due to binding

**Fig 4:**
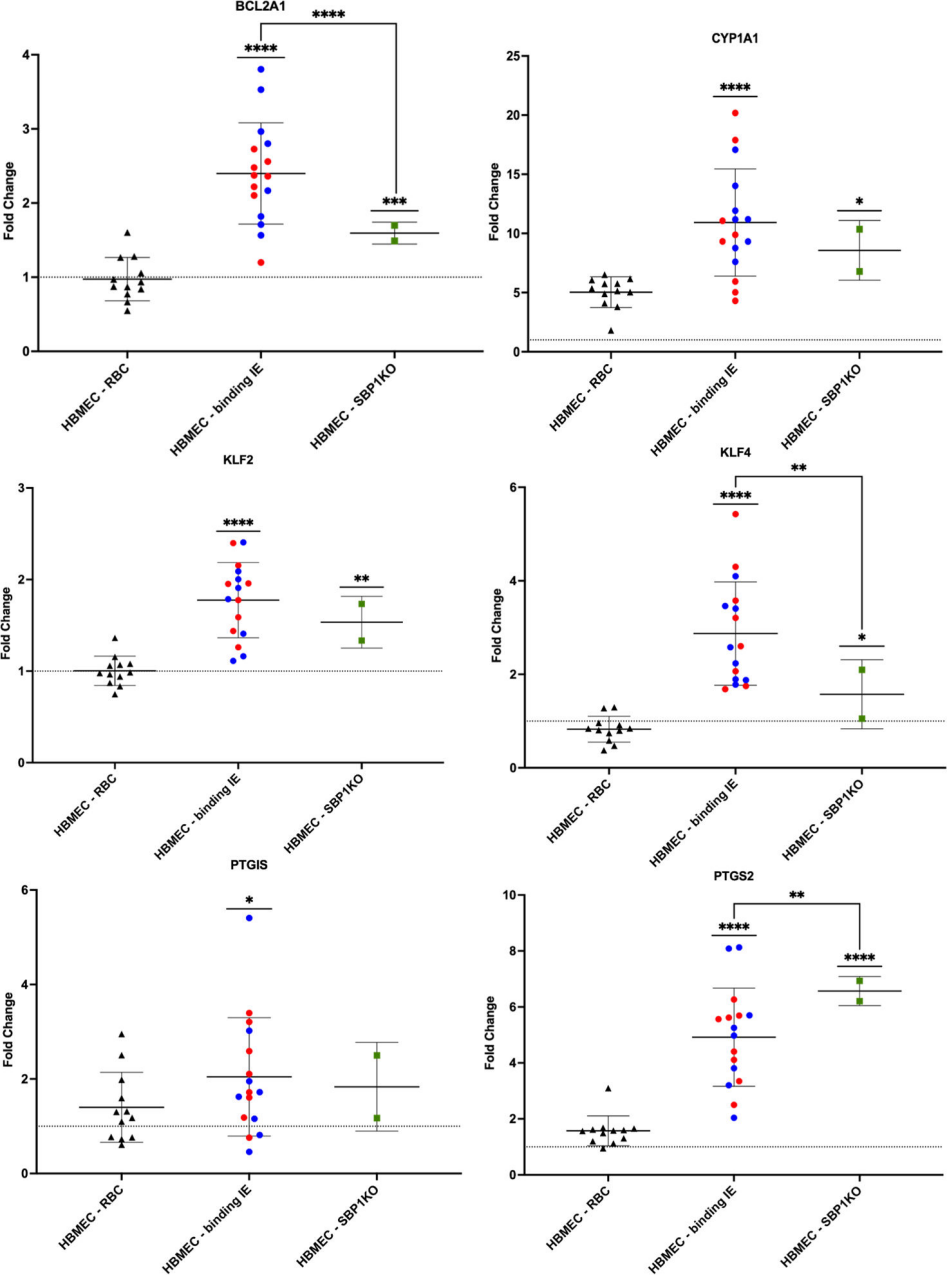
Observed differential effects on HBMEC transcriptional response are not due to binding to HBMECs. Relative gene expression of HBMECs exposed to patient-derived parasites that bind to HBMECs and the non-binding SBP1-KO parasites for 6 hours compared to corresponding RBCs for select genes. HBMEC-RBC samples for both binding and non-binding IE were combined for plotting. Each point on the plot represents one sample FC, calculated as a mean of the technical triplicates, and the mean and 95% confidence intervals are indicated for each group. FC was calculated using GAPDH as an endogenous control and relative to the 6-hour HBMEC-media normalization control (baseline is FC = 1). Welch’s *t*-tests were used to compare HBMEC-RBC and HBMEC-IE for each of the parasite groups, and the mean FC ± SEM for each gene was calculated using the two biological replicates for four UM and four CM isolates and their corresponding RBC [HBMEC-RBC (binding) *n* = 10, HBMEC-binding IE *n* = 16, HBMEC-RBC (SBP1KO) and HBMEC-SBP1KO (*n* = 2)]. The results of the Welch’s *t*-tests comparing the HBMEC-IE groups with corresponding HBMEC-RBC are summarized in Table S3. Sample grouping is represented by symbol, with triangles for HBMEC-RBC, circles for HBMEC-binding IE, and squares for HBMEC-SBP1KO. For binding IE, the red data points represent UM samples, while the blue data points represent CM samples. Samples from experiments with the non-binding parasites are in green. Significant relationships between HBMEC-IE groups and the corresponding HBMEC-RBC are indicated by straight bars over the IE groups (*P* < 0.05), and the *P*-value summaries are from the calculations performed in Table S3. For *BCL2A1*, *KLF4*, and *PTGS2*, the results of Welch’s *t*-tests comparing HBMEC-binding and HBMEC-SBP1KO are indicated by the bent lines connecting between these groups (*P* < 0.05), and the *P*-value summaries are from the calculations performed in Table S4. In all plots, the dotted line marks the baseline of FC = 1. *P*-value summary defined: *, 0.01–0.05; **, 0.001–0.01; ***, 0.0001–0.001; ****, <0.0001).

To further investigate whether the observed differential effects on HBMEC transcriptional responses were due to the direct interaction between the IE and the surface of HBMECs, two co-culture experiments were performed using the non-binding parasite strain, skeleton-binding protein knockout parasites. These parasites are unable to traffic *Pf*EMP1 to the infected erythrocyte surface, and therefore, they are unable to bind to endothelium (27, 34), verified by using flow binding assays to HBMECs (Fig. 3). The mean relative gene expression was calculated for the technical and biological replicates. Welch’s *t*-tests were performed to compare the relative FC of the HBMEC-SBP1-KO parasites and the HBMEC-RBC controls (Table S3). The relative gene expression of the HBMECs incubated with the binding patient-derived parasites (HBMEC-binding IE) and non-binding SBP1-KO parasites, as compared to the HBMEC-RBC controls, are shown in Fig. 4 for *BCL2A1*, *CYP1A1*, *KLF2*, *KLF4*, *PTGIS*, and *PTGS2*.

Even though similar effects were observed from the SBP1-KO parasites on HBMEC transcriptional response when compared to the patient-derived isolates for these genes, the magnitude of the effect is not always the same between HBMEC-SBP1-KO and the HBMEC-binding IE. The relative FC of the HBMEC-binding parasites and the HBMEC-SBP1-KO parasites were compared (Table S4), and out of the genes presented in Fig. 4, there were significant differences between the two groups for *BCL2A1*, *KLF4*, and *PTGS2*. There is large variation in responses within the binding IE group, likely due to the expression of different *Pf*EMP1 variants, suggesting that more than one process is occurring, one of which is adhesion independent. The observed differential effect on HBMEC transcriptional responses is related to parasite-infected RBC, but not exclusively binding via *Pf*EMP1.

As shown in Fig. 3, differences in cytoadhesion of the patient-derived isolates to HBMECs were measured after culturing. To determine *Pf*EMP1 domains expressed, *var* genotyping by qPCR was used and the data were compared to the qPCR data from when the parasites were isolated from the patients (Table S5) (26). Primer sets detecting multiple group A and A/B var domains were used, with the cysteine-rich interdomain region α1 (CIDRα1) domains predicting *Pf*EMP1 binding to EPCR and the duffy binding-like β (DBLβ) domains predicting binding to ICAM-1; both these receptors are expressed on HBMECs. A transcription unit of at least 32 denotes a transcription level equal to the endogenous genes seryl-tRNA synthetase and aldolase, used to normalize the data. High transcript levels of the initial DBLα domains remained for UM4 and CM1 after 26 and 29 DIC, respectively, while the levels decreased substantially for CM2 (43 DIC) and CM4 (33 DIC), indicative for *var* gene switching. CM1 seemed to maintain similar transcript levels for most of the var domains.

### Determination of prostaglandin endoperoxide synthase 2 and prostacyclin production in HBMEC-IE co-cultures

HBMEC-IE co-culture activates the prostacyclin pathway as shown for *PTGIS* and *PTGS2* in the Fluidigm results (Fig. 1). Whether that results in increased concentrations of its end-product prostacyclin or the intermediate PTGS2 was determined by enzyme-linked immunosorbent assay (ELISA). Prostacyclin is secreted but has a short half-life; therefore, its hydrolysis product, 6-keto prostaglandin F1α (6-keto PGF1α) was measured in co-culture medium of HBMEC-IE and compared with their respective HBMEC-RBC controls (Table S6; Fig. S3). Overall, there is some variability between the experiments, but no significant differences were detected between the co-cultures. However, the positive controls, HBMECs activated with TNF or thrombin, did increase 6-keto PGF1α concentrations. PTGS2 was detected in HBMEC cell lysate after co-culture and calculated as nanogram PTGS2 per milligram total lysate protein (Table S6). Overall, there was a small decrease in PTGS2 generation after HBMEC-IE co-culture, but only significant for CM1 (experiment 9) and CM4 (experiment 6).

### Production of cytokines by HBMEC-IE co-cultures

To determine if the production of cytokines or chemokines by HBMECs was altered following co-culture, a multiplex panel of 41 secreted cytokines and chemokines was used. The multiplex was performed at an early stage of the study and only included 6-hour co-culture medium of five isolates and two lab strains IT4var14 and IT4var37 in one experiment. These lab strains were included because they were used in previous co-culture experiments (25). Concentrations higher than 5 pg/mL were detected for 13 cytokines and only a few of these were significantly increased after co-culture with IE, compared to co-culture with their respective RBC control (Table S7). CM4, the isolate with the highest binding to HBMECs, significantly increased production of IL-6, IL-8, IFN-ɣ induced protein 10 (IP-10), growth-related oncogene (GRO), granulocyte colony-stimulating factor (G-CSF), and granulocyte-macrophage colony-stimulated factor (GM-CSF ). Although at low levels, IFNγ was increased 2- to 10-fold by co-culture with the patient isolates, but not significantly by the lab strains IT4var14 and IT4var37. A positive control of TNF-activated HBMECs was included to produce high enough levels of cytokines to be measured, and for 10 analytes, these were at least 10-fold higher than medium only. Notably, TNF was still detectable at ~11 ng/mL in the culture medium after 16 hours.

### Effect of IE co-culture on HBMEC barrier integrity

Although the cytoadherence phenotype of the isolates to HBMECs altered during culture, there are still detectable differences, especially between CM2 and CM4 with low and high binding, respectively. Whether differential binding capacity of the patient isolates could affect HBMEC barrier function was determined by *trans* endothelial electrical resistance (TEER). Barrier function increased slightly by adding RBC, irrespective if they were infected or not (Fig. S4B). Thrombin induces a rapid decrease in barrier function, which recovers within 2 hours. The presence of RBC and IE partly protected against this effect by reducing the maximum decrease (Fig. S4C and E) and the rate of recovery, determined by the area under the curve (AUC) (Fig. S4D and F). RBC and IE all significantly reduced thrombin-induced decrease in barrier function compared to medium, but IE was not significantly different to RBC. The recovery rate was more rapid with a significantly reduced AUC for RBC, 5%, 10%, and 30% IE compared to medium with a trend of more rapid recovery with increasing parasitemia. The AUCs for SBP1-KO at the different parasitemias were similar and not distinct compared to medium, but all significantly different than the AUC of 30% IE, which had the smallest AUC at 13.5. No differences between the individual UM- and CM-derived isolates could be detected.

## DISCUSSION

A view of CM pathology has emerged in recent years that proposes that dysregulation of coagulation pathways, inflammation, release of parasite factors from mature IE, and sequestration of IE in the vasculature all contribute to endothelial dysfunction and breakdown of the BBB (8, 9, 35). Cytoadhesion of IE to endothelial cells is one of the events in a multi-step process leading to localized accumulation of both IE and RBC, as well as rupture and release of parasite and intercellular erythrocyte components and soluble factors, resulting in activation of the endothelium and immune responses, compromising BBB integrity (20, 36).

In the present study, HBMEC responses to clinically derived parasites isolated from UM and CM patients were investigated by employing an *in vitro* co-culture model of cytoadhesion. We found that IE from both UM- and CM-derived isolates have differential effects on the HBMEC transcriptional response as compared to RBCs for 28 genes (Table S1; Fig. 1), but there was no difference when comparing UM with CM patient-derived isolates (Fig. 2). The UM- and CM-derived isolates were selected for this study based on their diverse binding capacities to HBMECs and HDMECs from a previous study (26), and their ability to be established in continuous culture to provide sufficient material for repeat experiments. Adapting *P. falciparum* patient-derived isolates to *in vitro* culture requires considerable time, and thus *var* gene switching is likely to occur (32, 37), which was the case for the CM and UM isolates, as detected by qPCR (Table S5). This resulted in altered binding profiles to TNF-stimulated HBMECs with less distinction between the isolates compared to that seen at the time of isolation from patients, with exceptions of CM2 and CM4, which still had relatively low and high binding to HBMECs, respectively (Fig. 3). Binding to HDMEC was not determined in this work for the cultured-adapted isolates, as the co-cultures were only performed with HBMECs. Despite the variability in binding profiles, we observed important differences in the HBMEC transcriptional response to IE compared to RBC in our *in vitro* model, supporting a role for *Pf*EMP1 in shaping the endothelial response to sequestration.

Comparing the binding capacity of each isolate to the relative gene expression of HBMECs after 6 hours co-culture detected no significant correlation between the parasites’ propensity to bind and the HBMEC gene expression of *PTGIS*, *PTGS2,* or *KL4* (Fig. S2). Furthermore, experiments performed using the SBP1-KO parasite line revealed that there were no differences in the HBMEC responses to binding and non-binding IE for these genes (Table S3), yet there was a significant difference in the magnitude of these responses between these two groups for 30 genes (Table S4). SBP1-KO parasites do not cytoadhere to HBMECs as they are unable to traffic *Pf*EMP1 to the surface, although they maintain both Maurer’s cleft and knob morphology and the expression of other Maurer’s cleft-associated proteins essential for tracking and IE surface changes, such as KAHRP, MAHRP, REX, *Pf*EMP3, and Pf332 (27, 34). RIFINs and STEVORs are proteins that are expressed on the surface of IE that are involved in cytoadherence to RBC (rosetting) and immune cells (38), and their trafficking and expression are maintained in SBP1-KO parasites (39). Possemiers et al. investigated sequestration deficiency in experimental *Plasmodium berghei* malaria-associated acute respiratory distress syndrome, and they observed endothelial activation in SBP1-KO-infected mice to the same level as wild type at 8 days post infection in the lungs (40). Overall, SBP1-KO IE maintain an altered surface, including knobs, and can interact with HBMECs differently compared to RBC. Our findings suggest that the interaction between HBMECs and IE via *Pf*EMP1 is not the only driver and that the observed differences in HBMEC relative gene expression are also related to contact with intact parasitized RBC.

We observed upregulation in relative gene expression of HBMECs after incubation with IE in a panel of genes, particularly in genes involved in apoptosis and the regulation of inflammation and endothelial integrity. Overall, the fold change expression levels were relatively low, except for the ones highlighted for further study and discussion. *BCL2A1* is a member of the pro-survival sub-family of BCL2 proteins that include both pro- and anti-apoptotic regulators, and it encodes a protein that reduces the release of pro-apoptotic cytochrome C and blocks caspase activation through inhibition of caspase-9 (41, 42). Expression of *BCL2A1* is upregulated in vascular endothelial cells by the inflammatory cytokines TNF and IL-1β (31, 43). *BCL2A1* expression was increased and a more robust *BCL2A1* expression was observed in HBMECs co-cultured with binding IE, as compared to non-binding SBP1-KO (Table S3). Perhaps, expression of *BLC2A1* early during an infection represents the balance between protection and pathology and demonstrates the temporal kinetics in this complicated system. KLF2 and KLF4 are transcription factors that have been shown to directly regulate endothelial function and integrity in vascular endothelium and to protect against endothelial dysfunction, both *in vitro* and in murine models, and their expression is modulated by both changes in laminar sheer flow or stress and pro-inflammatory stimuli (44–49). Overexpression of *KLF2* and *KLF4* affect coagulation and inflammatory pathways by inducing expression and activity of endothelial nitric oxide synthase and thrombomodulin (44–46, 50, 51) while simultaneously inhibiting expression of adhesion molecules E-selectin, ICAM-1, and VCAM-1 (45). We observed significant upregulation in relative gene expression of both *KLF2* and *KLF4* in HBMECs incubated with IE. CYP1A1, PTGIS, and PTGS2 are involved in lipid metabolism, including the biosynthesis of prostacyclin, which has roles as an inflammatory mediator and a potent vasodilator (52, 53). CYP1A1 is a member of the cytochrome P450 enzyme family that metabolizes a range of substrates, including arachidonic acid (54). Both PTGIS and PTGS2 are enzymes involved in the biosynthesis of prostacyclin, a bioactive lipid that is produced by vascular endothelial cells and that plays a role in regulation of endothelial inflammation and apoptosis (52). A recent study demonstrated differential gene expression of *CYP1A1*, *PTGIS*, and *PTGS2* between HBMECs incubated with two parasite lines that expressed different *Pf*EMP1s (25). As reported in that study, we observed evaluated levels of CYP1A1 induced by both IE and RBC controls. Despite observing transcriptional upregulation of *PTGIS* and *PTGS2*, FC ± SEM of 2.05 ± 0.24 and 4.92 ± 0.26 respectively (Table S3), this did not seem to translate into detection of PTGS2 protein or 6-keto PGF1α (prostacyclin’s stable hydrolysis product) in HBMEC lysate and co-culture supernatant, respectively (Table S6). PTGS2 is intracellularly localized, and expression in cell lysates is usually detected by Western blot. An ELISA was employed to quantify PTGS2 concentrations in multiple samples. Unfortunately, the usability of the ELISA kit was questionable, as the positive control, stimulation with TNF, did not result in increased PTGS2 production, and detection of PTGS2 was not pursued further. 6-Keto PGF1α is secreted, and larger amounts were produced upon stimulation with TNF and thrombin, but overall, no significant increase in production was detected for the HBMEC-IE co-cultures compared to their respective HBMEC-RBC co-cultures. Overall, our findings provide additional evidence that IE regulate brain ECs and that these effects are not only due directly to binding.

Previous studies have investigated the effects of IE on endothelial responses *in vitro*. An investigation by Zuniga et al. examined how TNF and IE differently affected transcriptional responses and barrier integrity in brain endothelial cells. Their work highlighted that TNF and IE lysates induce distinct transcriptional profiles, with TNF associated with induction of endothelial activation while IE lysate contributed considerably to endothelial barrier disruption, yet overlap in genes associated with inflammation, including induction of PTGS2, was detected (23). Howard et al. adapted a 3D perfusion human brain microvessel model to evaluate the brain endothelial responses to perfusion with TNF and at different stages of parasite maturation. This study found that treatment of brain microvessels with TNF induced an inflammatory phenotype, while EPCR-binding parasites caused localized barrier damage, and induced unique stress response pathways associated with metal toxicity and oxidative stress (30). In concordance with Zuniga et al. and Howard et al. (23, 30), we observed that a group of inflammatory-associated genes is upregulated in HBMECs by exposure to patient-derived IE and this effect is independent of TNF treatment. This upregulation in HBMEC relative gene expression was detected after 6-hour incubation with trophozoite-stage IE, in contrast to studies that used schizont-stage IE or IE lysates for longer periods ranging from 6, 9, 12, and 24 hours (23, 30). In contrast to these studies (23, 30, 55), we utilized trophozoite-IE prepared in EGM2min for a 6-hour incubation period. This medium maintains HBMECs over the time course, but affects the progress of IE, as they barely develop beyond the trophozoite stage. In our study, we detect HBMEC effects at early stages of sequestration where IE are still in contact with the endothelium, as opposed to effects attributed to parasite material and ruptured IE. An extended co-culture time frame would have allowed us to observe other, perhaps larger, effects on HBMEC transcription. This agrees with the proposed multi-step activation of endothelium in which binding results in a high local concentration of soluble erythrocytic and parasitic factors (20).

A multiplex assay was used to detect 41 cytokines in the co-culture supernatant. HBMECs produce low levels of cytokines in their basal state, and this has not much increased after co-culture with IE, with a relatively low FC comparing IE to RBC co-culture (Table S7). The multiplex was not performed with all the isolates nor SBP1-KO, and only with one experiment; thus, the results are only an indication of the effects of IE co-culture. Stimulation with TNF, as positive control, did increase the concentrations of multiple cytokines, including IL-6, IL-8, IP-10,and monocyte chemoattractant protein-1 (MCP-1), also reported in other studies (23, 55, 56). Indeed, Zuniga et al. (23) noted they observed transcriptionally similar but translationally different levels of cytokines between their treatment groups even when total protein synthesis levels were similar, suggesting a role for post-translational regulation in HBMECs after incubation with IE.

Whether the patient-derived isolates affect HBMEC barrier integrity was determined by TEER, with cell index as measure of integrity. In all experiments, the cell index was increased after adding IE, but to a similar level as RBC, likely to be due to the layer of RBC on top of the HBMECs affecting conductivity (Fig. S4A and B). None of the isolates decreased the HBMEC barrier function, even after 24 hours (Fig. S4B). This contrasts with other studies where the addition of IE decreased the barrier integrity (23, 55) and is likely related to their use of schizont-stage IE or IE lysates, while trophozoite-stage IE were used in our experiments. After 24 hours, parasites seemed to be in a resting stage and were not progressing to schizont stage, therefore not releasing parasite factors, previously identified as the reason for the decrease in barrier function (23, 55, 57).

It has been reported that IE affect the endothelial responses to thrombin (23, 55), which reduces barrier function rapidly and quickly recovers. The presence of RBC or IE affected the response of HBMECs to thrombin with a reduced maximum response and AUC, compared to medium (Fig. S4E and F). There were no significant differences between the effect of RBC or IE, but the non-binding lab isolates SBP1-KO, at the three different parasitemias, was significantly different to the effect of 30% IE (Fig. S4D through F), indicating that receptor binding may be required to reduce the effect of thrombin. In contrast, Avril et al. showed that schizont IE, but not trophozoite IE, prolonged thrombin-induced barrier disruption, compared to RBC (55). However, thrombin was added 2–3 hours after adding IE or RBC and not compared to the effect of thrombin in medium. Thrombin decreases endothelial barrier function by cleavage of protease-activated receptor 1 (PAR1) and involves the thrombomodulin/activated protein C system in which the balance of thrombin and activated protein C determines PAR1-dependent barrier disruptive or protective action, respectively (14, 58). The reduced effect of thrombin indicates a shift toward more activated protein C, which was not investigated in this study. However, binding of IE to HBMECs induces receptor shedding and this could change the level of thrombomodulin and endothelial protein C receptor, the receptors for thrombin and protein C, respectively (57, 59), affecting PAR1 cleavage. The TEER data show that specific binding of IE to HBMECs do affect certain endothelial responses, not captured in the RNA transcription data.

One limitation of this study was the use of a co-culture model that simplified the complex microvascular system down to static interaction between IE and HBMECs. Nonetheless, this model allowed for the study of the HBMEC transcriptional response to patient-derived parasites and to assess if these responses were dependent on IE cytoadhesion or clinical syndrome. The ratio of 110 IE/HBMECs in our experiments is similar to the range employed in other studies (23, 55), which was calculated in Zuniga et al. to be equivalent to approximately two layers of IE on top of the HBMECs. We tested whether the Fluidigm panel would reflect endothelial gene expression changes by exposing HBMECs overnight to IL-1β and TNF and comparing the gene expression with a media control (Table S2). We observed massive upregulation in HBMEC expression of several genes, including *CXCL3*, *ICAM1*, *PTGS2*, *SELE*, and *VCAM1*, after incubation with both stimuli, confirming that our model was able to determine regulation of specific genes. Furthermore, there was significant reduction in relative gene expression after withdrawal of TNF from HBMECs (Fig. S1). Overall, these findings suggest that the changes in relative gene expression observed in HBMECs exposed to both binding and non-binding parasites are due to the impact of IE.

During early stages of sequestration, we observed effects on endothelium that are both dependent and independent of *Pf*EMP1. These effects were relatively small except for several genes, including *CYP1A1*, *KLF4*, and *PTGS2*. These genes may represent early endothelial protective responses that act through vascular regulation and modulation of thrombomodulin. There was no difference in gene regulation in HBMECs based on the origin of the isolate nor the parasite-binding capacities. Overall, interaction of IE and endothelial cells in early stages of sequestration does induce some endothelial responses, including some that are independent of P*f*EMP1, and this is the first investigation to our knowledge to study how non-*Pf*EMP1 expressing IE affects endothelial transcriptional responses. Understanding the different mechanisms of crosstalk between *P. falciparum*-infected erythrocytes and host endothelium may help us to develop interventions that support patients with severe malaria while effective anti-parasite drugs clear the infection.

## Data Availability

All data are contained in the article and supplementary files.

## SUPPLEMENTAL MATERIAL

Supplemental material spectrum.00727-24-s0001.pdf Tables S1 to S7; Fig. S1 to S4.

Supplemental data spectrum.00727-24-s0002.xlsx Real-Time Data from the BioMark HD Instrument.

ASM does not own the copyrights to Supplemental Material that may be linked to, or accessed through, an article. The authors have granted ASM a non-exclusive, world-wide license to publish the Supplemental Material files. Please contact the corresponding author directly for reuse.
