# Supplementary material for: Transcriptional responses of brain endothelium to Plasmodium falciparum patient-derived isolates in vitro
Source: Microbiol Spectr. 2024 Jun 12;12(7):e00727-24. doi: 10.1128/spectrum.00727-24 (PMC11218514; doi:10.1128/spectrum.00727-24)
Supplement: Supplemental material — Tables S1 to S7; Fig. S1 to S4. [file spectrum.00727-24-s0001.pdf]

Supplementary Data

Supplementary Table 1:* Relative gene expression of HBMEC co-cultured with IE from patient-derived isolates or with the corresponding RBC control for each gene in the panel.** Samples were collected after a 6-hour co-culture, and relative gene expression was calculated using the $\Delta\Delta$Ct method using GAPDH as the endogenous control and the corresponding experimental 6-hour HBMEC-media sample as the reference sample (baseline is FC =1). The mean Fold Change (FC) and standard error of the mean (SEM) for each gene were calculated using the technical triplicates of the two biological replicates (UM = uncomplicated malaria patient-derived isolates, CM = cerebral malaria patient-derived isolates, n = sample size). Rows shaded in dark grey have FC>1.5 for one of the IE groups. P-value summary defined= *,0.01-0.05; **, 0.001-0.01; ***, 0.0001-0.001; *, <0.0001.

	**WELCH'S T-TEST COMPARING HBMEC-RBC (N=21) AND HBMEC-UM (N=24) RELATIVE FC**				**WELCH'S T-TEST COMPARING HBMEC-RBC (N=21) AND HBMEC-CM (N=24) RELATIVE FC**			
**GENE**	HBMEC-RBC(UM) mean FC ± SEM	HBMEC-UM mean FC ± SEM	P value	P value summary	HBMEC-RBC(CM) mean FC ± SEM	HBMEC-CM mean FC ± SEM	P value	P value summary
ACTB	1.01 ± 0.026	1.12 ± 0.053	0.0039	**	0.94 ± 0.02	1.23 ± 0.047	<0.0001	****
ACTG1	1.03 ± 0.028	1.25 ± 0.033	<0.0001	****	1.01 ± 0.021	1.29 ± 0.046	<0.0001	****
ADAM10	1.01 ± 0.016	1.16 ± 0.031	0.0002	***	1 ± 0.021	1.13 ± 0.028	0.0012	**
ADAM17	0.92 ± 0.04	1.14 ± 0.025	<0.0001	****	0.91 ± 0.027	1.1 ± 0.04	0.0002	***
ANGTP2	1.05 ± 0.037	0.9 ± 0.025	0.0014	**	1.09 ± 0.021	0.96 ± 0.043	0.0075	**
ANK1	0.84 ± 0.049	0.99 ± 0.087	0.1365	ns	0.91 ± 0.086	1.22 ± 0.17	0.1132	ns
BAX	0.97 ± 0.028	0.98 ± 0.031	0.8577	ns	0.97 ± 0.022	1 ± 0.024	0.2942	ns
BCL2A1	1.03 ± 0.078	2.25 ± 0.093	<0.0001	****	1.03 ± 0.064	2.55 ± 0.171	<0.0001	****
CASP3	1.05 ± 0.041	1.07 ± 0.064	0.7753	ns	1.01 ± 0.03	0.98 ± 0.041	0.6767	ns
CCL14	0.96 ± 0.038	0.67 ± 0.035	<0.0001	****	1 ± 0.039	0.78 ± 0.031	0.0001	***
CDH5	0.99 ± 0.022	1.02 ± 0.022	0.3607	ns	0.96 ± 0.024	1 ± 0.013	0.1881	ns
CXCL3	0.79 ± 0.106	1.82 ± 0.26	0.0009	***	0.86 ± 0.097	1.72 ± 0.184	0.0002	***
CYP1A1	4.6 ± 0.374	10.46 ± 1.388	0.0004	***	5.32 ± 0.299	11.39 ± 0.838	<0.0001	****
DUSP5	0.96 ± 0.032	1.52 ± 0.048	<0.0001	****	0.91 ± 0.045	1.44 ± 0.069	<0.0001	****
EEF1A1	1.01 ± 0.056	1.21 ± 0.058	0.0195	*	0.97 ± 0.04	1.22 ± 0.06	0.0012	**
FAS	0.96 ± 0.058	0.96 ± 0.072	0.9836	ns	1.02 ± 0.057	0.94 ± 0.048	0.302	ns
HES1	1.05 ± 0.043	1.32 ± 0.075	0.004	**	1.02 ± 0.045	1.28 ± 0.088	0.0135	*
HEY1	0.81 ± 0.055	0.84 ± 0.054	0.7524	ns	0.73 ± 0.045	0.74 ± 0.048	0.8462	ns
HEY2	1.38 ± 0.123	1.24 ± 0.118	0.4226	ns	1.43 ± 0.116	1.38 ± 0.138	0.7798	ns
ICAM1	1 ± 0.109	1.39 ± 0.144	0.0358	*	0.97 ± 0.109	1.41 ± 0.116	0.0088	**
IL1A	1.21 ± 0.085	1.92 ± 0.106	<0.0001	****	1.27 ± 0.084	1.99 ± 0.112	<0.0001	****
IL6	1.16 ± 0.062	1.39 ± 0.053	0.0079	**	1.01 ± 0.033	1.59 ± 0.103	<0.0001	****
KLF10	1.04 ± 0.031	1.17 ± 0.071	0.0986	ns	0.97 ± 0.037	1.02 ± 0.057	0.4834	ns
KLF2	1.03 ± 0.039	1.82 ± 0.079	<0.0001	****	0.96 ± 0.029	1.73 ± 0.096	<0.0001	****
KLF3	0.93 ± 0.065	1.02 ± 0.033	0.2563	ns	0.89 ± 0.039	1 ± 0.031	0.0452	*
KLF4	0.86 ± 0.089	3.08 ± 0.339	<0.0001	****	0.77 ± 0.061	2.67 ± 0.208	<0.0001	****
LAMC2	1 ± 0.034	1.14 ± 0.034	0.0039	**	0.98 ± 0.038	1.2 ± 0.051	0.0016	**
MIB1	1.09 ± 0.034	1.31 ± 0.047	0.0005	***	0.98 ± 0.039	1.26 ± 0.046	<0.0001	****
NFKB1	1 ± 0.039	1.1 ± 0.059	0.1724	ns	0.93 ± 0.031	0.94 ± 0.038	0.8053	ns
NFKBIA	0.81 ± 0.057	0.9 ± 0.053	0.2358	ns	0.82 ± 0.057	0.87 ± 0.045	0.4546	ns

NOTCH2	1.04 ± 0.104	1.16 ± 0.105	0.4248	ns	1.03 ± 0.109	1.48 ± 0.197	0.054	ns
OCLN	0.97 ± 0.081	0.87 ± 0.072	0.3534	ns	0.9 ± 0.061	1.02 ± 0.047	0.1321	ns
PAPLN	1.1 ± 0.082	1.53 ± 0.095	0.0013	**	1.07 ± 0.073	1.47 ± 0.127	0.01	**
PECAM1	0.92 ± 0.027	1.08 ± 0.033	0.0006	***	0.92 ± 0.019	1.07 ± 0.024	<0.0001	****
PLA2G4A	1.23 ± 0.071	1.49 ± 0.095	0.0336	*	1.04 ± 0.06	1.3 ± 0.078	0.0113	*
PLXNA4	1.44 ± 0.318	1.37 ± 0.308	0.8739	ns	1.24 ± 0.314	1.35 ± 0.285	0.8057	ns
PROCR	1.03 ± 0.024	1.22 ± 0.04	0.0002	***	0.99 ± 0.029	1.25 ± 0.052	<0.0001	****
PTGIS	1.04 ± 0.122	2.07 ± 0.284	0.0022	**	1.52 ± 0.255	2.02 ± 0.391	0.2894	ns
PTGS2	1.54 ± 0.153	4.69 ± 0.275	<0.0001	****	1.51 ± 0.048	5.15 ± 0.431	<0.0001	****
S100A10	0.92 ± 0.026	1 ± 0.029	0.0526	ns	0.94 ± 0.019	1.04 ± 0.019	0.0007	***
SELE	0.72 ± 0.111	0.78 ± 0.111	0.7099	ns	0.87 ± 0.125	1.46 ± 0.135	0.0023	**
SMAD6	1.4 ± 0.099	1.66 ± 0.125	0.1028	ns	1.14 ± 0.097	1.82 ± 0.178	0.0019	**
TGFB1	1 ± 0.033	1.17 ± 0.045	0.0039	**	0.94 ± 0.027	1.11 ± 0.039	0.0013	**
TJP1	0.99 ± 0.031	0.93 ± 0.032	0.1611	ns	0.9 ± 0.031	0.93 ± 0.03	0.4792	ns
TNFRSF12A	1.31 ± 0.084	1.3 ± 0.147	0.9386	ns	1.14 ± 0.105	1.08 ± 0.089	0.626	ns
TSPAN13	0.96 ± 0.052	1.1 ± 0.052	0.0645	ns	0.93 ± 0.049	1.06 ± 0.04	0.0457	*
TUBB	1.02 ± 0.02	1.02 ± 0.026	0.9383	ns	0.98 ± 0.016	1.05 ± 0.02	0.0115	*
TXNIP	1.47 ± 0.165	2.63 ± 0.485	0.0311	*	0.98 ± 0.054	2.9 ± 0.64	0.0064	**
VCAM1	0.83 ± 0.083	1.78 ± 0.121	<0.0001	****	0.85 ± 0.087	2.06 ± 0.252	<0.0001	****

Supplementary Table 2: **Assessment of endothelial responses and Fluidigm panel through relative gene expression of HBMEC after overnight incubation with inflammatory cytokines**. HBMEC were incubated overnight with either EGM2min media, 1 ng/mL IL-1beta, or 10 ng/mL TNF. The samples were collected directly after stimulation, and the relative gene expression was determined for several genes. FCs were calculated using GAPDH as an endogenous control as relative to the HBMEC-overnight media normalization control (baseline is FC=1). The mean FC for each gene was calculated from technical triplicates from one experiment (n=3).

GENE	HBMEC-OVN MEDIA RELATIVE FC	HBMEC OVN IL-1B RELATIVE FC	HBMEC-OVN TNF RELATIVE FC
BCLA2A1	1	4.7	6.0
CXCL3	1	1305	862
CYP1A1	1	1.1	0.6
ICAM1	1	16	25
IL1A	1	40	30
IL1B	1	60	5.4
KLF2	1	1.1	1.0
KLF4	1	3.1	2.9
PTGIS	1	0.8	1.2
PTGS2	1	30	16
SELE	1	72	52
VCAM1	1	548	873

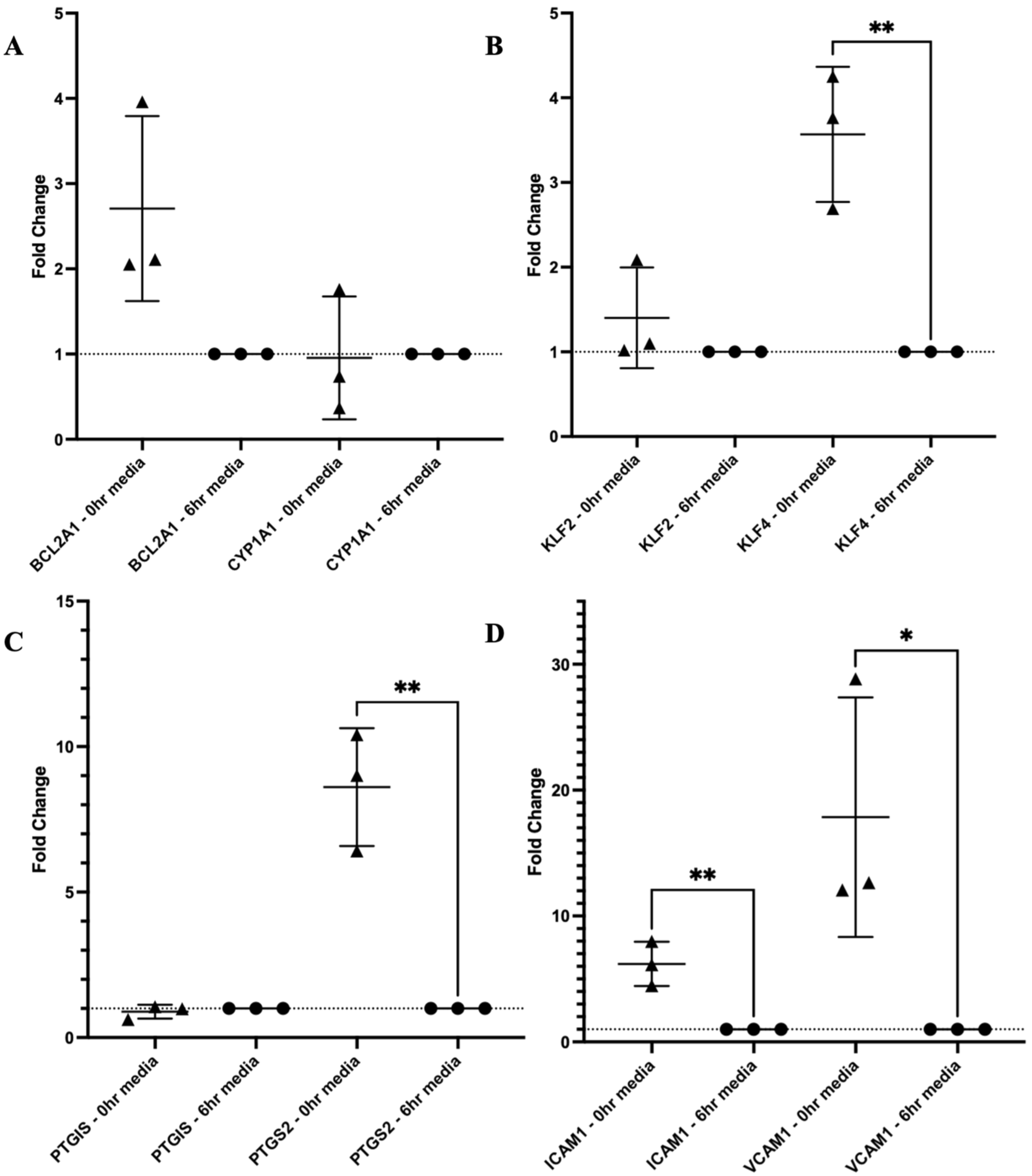

Supplementary Figure 1: **Representative relative gene expression of HBMEC after withdrawal of TNF stimulation.** In the co-culture model, HBMEC are stimulated overnight with 10 ng/mL of TNF to stimulate the cells. The next morning, the supernatant containing TNF was removed and replaced with EGM2min media for two hours before co-culture experiments commenced. HBMEC-media samples were collected at 0hr and 6hour culture time points, corresponding to 2- and 8-hours incubation with EGM2min media and without TNF, respectively. Relative gene expression was determined for (A) *BCL2A1* and *CYP1A1*, (B) *KLF2* and *KLF4*, (C) *PTGIS* and *PTGS2*, and (D) *ICAM-1* and *VCAM1* for HBMEC-media samples for three independent, representative experiments (n=3). FCs were calculated using GAPDH as an endogenous control as relative to the HBMEC-6hour media normalization control (baseline is FC=1). Each plot point represents a mean of the technical triplicates, and the mean ± SD of the 3 experiments is shown with triangle points representing HBMEC-0hr media samples and circular points representing HBMEC-6hr media samples.

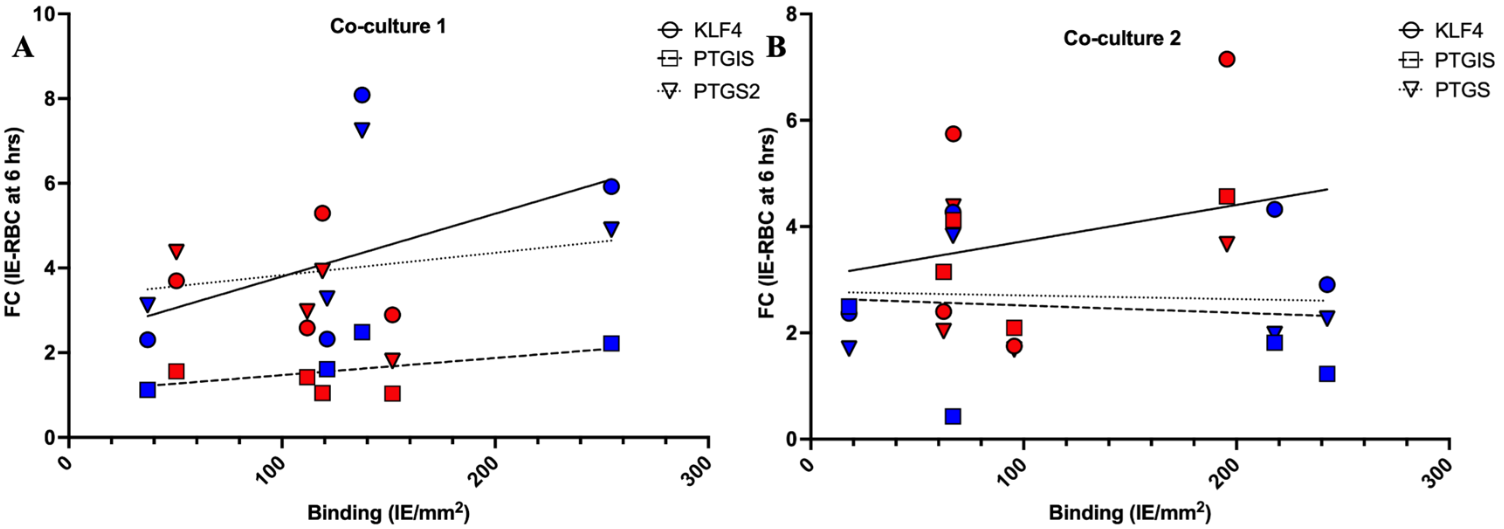

Supplementary Figure 2: **Binding capacities of patient-derived parasite isolates do not correlate with HBMEC gene expression.** The binding capacity of each isolate (IE/ mm^2) was compared to the 6-hour HBMEC mean FC relative gene expression for select genes (red = UM isolates; blue = CM isolates) after the first (A) and second (B) experiments using a linear regression. The linear regression for KLF4, PTGIS, and PTGS2 are indicated on each graph by straight, dashed, and dotted lines, respectively, and the R^2 values are reported as follows: Co-culture 1) KLF4 = 0.22, PTGIS= 0.25, PTGS2= 0.05; Co-culture 2) KLF4 = 0.10, PTGIS= 0.01, PTGS2= 0.003. Relative gene expression was calculated for HBMEC-IE using the ΔΔCt method with GAPDH as the endogenous control and the corresponding 6-hour HBMEC-RBC as the reference sample (baseline is FC=1). Each point represents the mean FC for each HBMEC-IE sample (n=8), calculated from the technical triplicates. Pearson's correlation was performed to compare parasite binding capacity to the differential gene expression for each isolate for both experiments, and the correlation coefficient (r) values are reported as follows: Co-culture 1) KLF4 = 0.47, PTGIS= 0.50, PTGS2= 0.22; Co-culture 2) KLF4 = 0.31, PTGIS= -0.08, PTGS2= -0.05.

Supplementary Table 3: **Relative gene expression of HBMEC co-cultured with either binding IE or non-binding IE compared to the corresponding HBMEC-RBC control for each gene in the panel.** All samples were from a 6-hour time point. Data from UM and CM patient-derived parasite isolates were combined to form a group of binding parasites, while two experiments with non-binding SBP1KO parasites were performed. Relative gene expression was calculated using the ΔΔCt method using GAPDH as the endogenous control and the corresponding experimental 6-hour HBMEC-media sample as the reference sample (baseline is FC=1). The mean Fold Change (FC) and standard error of the mean (SEM) for each gene were calculated using all experimental samples and technical replicates (SBP1KO= SBP1-knockout parasites, n = sample size). Rows shaded in dark grey have FC>1.5 for one of the HBMEC groups. P-value summary defined= *,0.01-0.05; **, 0.001-0.01; ***, 0.0001-0.001; ****, <0.0001.

	**WELCH'S T-TEST COMPARING HBMEC-RBC (N=30) AND HBMEC-BINDING (N=48) RELATIVE FC**				**WELCH'S T-TEST COMPARING HBMEC-RBC (N=6) AND HBMEC-SBP1KO (N=6) RELATIVE FC**			
**GENE**	HBMEC-RBC mean FC ± SEM	HBMEC-binding mean FC ± SEM	P value	P value summary	HBMEC-RBC mean FC ± SEM	HBMEC-SBP1KO mean FC ± SEM	P value	P value summary
ACTB	0.97 ± 0.022	1.21 ± 0.035	<0.0001	****	0.96 ± 0.035	1.06 ± 0.02	0.0372	*
ACTG1	1.01 ± 0.021	1.27 ± 0.028	<0.0001	****	0.95 ± 0.035	0.98 ± 0.032	0.6649	ns
ADAM10	1.01 ± 0.016	1.14 ± 0.021	<0.0001	****	0.88 ± 0.044	1.03 ± 0.04	0.0299	*
ADAM17	0.93 ± 0.03	1.12 ± 0.023	<0.0001	****	0.87 ± 0.036	1.05 ± 0.033	0.0045	**
ANGTP2	1.06 ± 0.027	0.93 ± 0.025	0.0007	***	1.01 1.01 ± 0.025	0.94 ± 0.04	0.1918	ns
ANK1	0.88 ± 0.065	1.1 ± 0.096	0.0535	ns	0.83 ± 0.184	0.83 ± 0.098	0.9934	ns

BAX	0.98 ± 0.02	0.99 ± 0.019	0.7438	ns	0.96 ± 0.05	0.81 ± 0.039	0.0424	*
BCL2A1	0.97 ± 0.057	2.4 ± 0.099	<0.0001	****	1.01 ± 0.078	1.59 ± 0.058	0.0002	***
CASP3	1.02 ± 0.033	1.03 ± 0.038	0.8538	ns	0.94 ± 0.056	0.87 ± 0.059	0.3719	ns
CCL14	0.96 ± 0.031	0.73 ± 0.025	<0.0001	****	0.75 ± 0.06	0.48 ± 0.022	0.0052	**
CDH5	0.96 ± 0.019	1.01 ± 0.013	0.0399	*	0.98 ± 0.023	0.88 ± 0.016	0.0075	**
CXCL3	0.8 ± 0.075	1.77 ± 0.157	<0.0001	****	2.17 ± 0.371	2.89 ± 0.551	0.3076	ns
CYP1A1	4.94 ± 0.314	10.92 ± 0.805	<0.0001	****	5.54 ± 0.604	8.57 ± 1.017	0.0329	*
DUSP5	0.9 ± 0.033	1.48 ± 0.042	<0.0001	****	1.06 ± 0.111	2.11 ± 0.138	0.0002	***
EEF1A1	0.97 ± 0.042	1.22 ± 0.041	<0.0001	****	0.89 ± 0.06	0.84 ± 0.023	0.4657	ns
FAS	1 ± 0.05	0.95 ± 0.043	0.4783	ns	0.75 ± 0.079	0.69 ± 0.062	0.6216	ns
HES1	1.01 ± 0.034	1.3 ± 0.057	<0.0001	****	0.9 ± 0.036	1.47 ± 0.056	<0.0001	****
HEY1	0.79 ± 0.046	0.79 ± 0.037	0.8966	ns	0.76 ± 0.088	0.62 ± 0.04	0.1779	ns
HEY2	1.32 ± 0.095	1.31 ± 0.09	0.9267	ns	0.91 ± 0.202	1.47 ± 0.359	0.2132	ns
ICAM1	1 ± 0.08	1.4 ± 0.091	0.0016	**	1.24 ± 0.118	1.73 ± 0.293	0.1706	ns
IL1A	1.23 ± 0.063	1.95 ± 0.076	<0.0001	****	1.5 ± 0.095	1.91 ± 0.177	0.077	ns
IL6	1.11 ± 0.048	1.49 ± 0.059	<0.0001	****	1.32 ± 0.085	1.7 ± 0.149	0.0551	ns
KLF10	0.98 ± 0.029	1.09 ± 0.046	0.048	*	1.18 ± 0.112	1.3 ± 0.108	0.4584	ns
KLF2	1 ± 0.032	1.78 ± 0.062	<0.0001	****	1.01 ± 0.073	1.53 ± 0.12	0.0056	**
KLF3	0.94 ± 0.046	1.01 ± 0.022	0.1811	ns	0.9 ± 0.042	0.7 ± 0.032	0.005	**
KLF4	0.84 ± 0.069	2.87 ± 0.199	<0.0001	****	0.77 ± 0.098	1.58 ± 0.254	0.0239	*
LAMC2	0.99 ± 0.029	1.17 ± 0.031	<0.0001	****	1.12 ± 0.093	1.24 ± 0.142	0.482	ns
MIB1	1.04 ± 0.033	1.29 ± 0.033	<0.0001	****	0.73 ± 0.052	1.16 ± 0.046	0.0001	***
NFKB1	0.98 ± 0.03	1.02 ± 0.037	0.445	ns	0.79 ± 0.062	0.7 ± 0.059	0.2969	ns
NFKBIA	0.82 ± 0.041	0.89 ± 0.034	0.2302	ns	1.16 ± 0.085	1.13 ± 0.102	0.8297	ns
NOTCH2	1.05 ± 0.089	1.32 ± 0.113	0.0702	ns	1.6 ± 0.821	2.62 ± 1.152	0.4893	ns
OCLN	0.95 ± 0.058	0.94 ± 0.044	0.9851	ns	0.88 ± 0.084	0.92 ± 0.095	0.7435	ns
PAPLN	1.04 ± 0.065	1.5 ± 0.079	<0.0001	****	0.95 ± 0.149	1 ± 0.083	0.7714	ns
PECAM1	0.91 ± 0.02	1.07 ± 0.02	<0.0001	****	0.96 ± 0.034	0.91 ± 0.032	0.2936	ns
PLA2G4A	1.15 ± 0.057	1.4 ± 0.062	0.0046	**	1.31 ± 0.224	1.51 ± 0.173	0.4987	ns
PLXNA4	1.32 ± 0.225	1.36 ± 0.207	0.9008	ns	1.01 ± 0.267	0.58 ± 0.068	0.1718	ns
PROCR	0.99 ± 0.022	1.24 ± 0.033	<0.0001	****	0.89 ± 0.037	0.92 ± 0.033	0.5743	ns
PTGIS	1.35 ± 0.188	2.05 ± 0.239	0.0252	*	1.64 ± 0.367	1.84 ± 0.442	0.7465	ns
PTGS2	1.57 ± 0.107	4.92 ± 0.255	<0.0001	****	1.58 ± 0.144	6.57 ± 0.422	<0.0001	****
S100A10	0.93 ± 0.02	1.02 ± 0.017	0.001	**	0.93 ± 0.053	0.82 ± 0.045	0.1704	ns
SELE	0.88 ± 0.1	1.12 ± 0.1	0.0935	ns	1.45 ± 0.286	2.66 ± 0.298	0.0147	*
SMAD6	1.25 ± 0.082	1.74 ± 0.108	0.0005	***	1.74 ± 0.381	1.52 ± 0.178	0.6283	ns
TGFB1	0.97 ± 0.025	1.14 ± 0.03	<0.0001	****	0.93 ± 0.042	1.14 ± 0.065	0.0236	*
TJP1	0.94 ± 0.028	0.93 ± 0.022	0.7211	ns	0.85 ± 0.069	0.77 ± 0.048	0.3735	ns
TNFRSF12A	1.24 ± 0.087	1.19 ± 0.087	0.6808	ns	1.2 ± 0.184	0.84 ± 0.171	0.1886	ns
TSPAN13	0.93 ± 0.038	1.08 ± 0.033	0.0051	**	0.86 ± 0.044	0.84 ± 0.033	0.6971	ns
TUBB	1.01 ± 0.017	1.04 ± 0.016	0.2726	ns	0.88 ± 0.044	0.9 ± 0.036	0.8362	ns

TXNIP	1.29 ± 0.129	2.76 ± 0.397	0.0008	***	0.81 ± 0.11	0.98 ± 0.18	0.4599	ns
VCAM1	0.84 ± 0.065	1.92 ± 0.14	<0.0001	****	1.13 ± 0.067	3.84 ± 0.482	0.0023	**

Supplementary Table 4: **Comparing the relative gene expression of HBMEC co-cultured with either binding IE or non-binding IE for each gene in the panel.** Welch's T-test was performed to compare the mean FC expression of HBMEC incubated with either binding or no-binding IE. All samples were from a 6-hour time point. Data from UM and CM patient-derived parasite isolates were combined to form a group of binding parasites, while two experiments with non-binding SBP1KO parasites were performed. Relative gene expression was calculated using the ΔΔCt method using GAPDH as the endogenous control and the corresponding experimental 6-hour HBMEC-media sample as the reference sample (baseline is FC=1). The mean Fold Change (FC) and standard error of the mean (SEM) for each gene were calculated using all experimental samples and technical replicates (SBP1KO= SBP1-knockout parasites, n = sample size). Rows shaded in dark grey have FC>1.5 for one of the HBMEC groups. P-value summary defined= *,0.01-0.05; **, 0.001-0.01; ***, 0.0001-0.001; ****, <0.0001.

WELCH'S T-TEST COMPARING HBMEC-BINDING (N=48) AND HBMEC-SBP1KO (N=6) RELATIVE FC

**GENE**	HBMEC-binding mean FC ± SEM	HBMEC-SBP1KO mean FC ± SEM	P value	P value summary
ACTB	1.21 ± 0.035	1.06 ± 0.02	0.0004	***
ACTG1	1.27 ± 0.028	0.98 ± 0.032	<0.0001	****
ADAM10	1.14 ± 0.021	1.03 ± 0.04	0.0366	*
ADAM17	1.12 ± 0.023	1.05 ± 0.033	0.1025	ns
ANGTP2	0.93 ± 0.025	0.94 ± 0.04	0.7908	ns
ANK1	1.1 ± 0.096	0.83 ± 0.098	0.0647	ns
BAX	0.99 ± 0.019	0.81 ± 0.039	0.0039	**
BCL2A1	2.4 ± 0.099	1.59 ± 0.058	<0.0001	****
CASP3	1.03 ± 0.038	0.87 ± 0.059	0.0452	*
CCL14	0.73 ± 0.025	0.48 ± 0.022	<0.0001	****
CDH5	1.01 ± 0.013	0.88 ± 0.016	<0.0001	****
CXCL3	1.77 ± 0.157	2.89 ± 0.551	0.0993	ns
CYP1A1	10.92 ± 0.805	8.57 ± 1.017	0.0935	ns
DUSP5	1.48 ± 0.042	2.11 ± 0.138	0.0047	**
EEF1A1	1.22 ± 0.041	0.84 ± 0.023	<0.0001	****
FAS	0.95 ± 0.043	0.69 ± 0.062	0.0066	**
HES1	1.3 ± 0.057	1.47 ± 0.056	0.0401	*
HEY1	0.79 ± 0.037	0.62 ± 0.04	0.0062	**
HEY2	1.31 ± 0.09	1.47 ± 0.359	0.6924	ns
ICAM1	1.4 ± 0.091	1.73 ± 0.293	0.3252	ns
IL1A	1.95 ± 0.076	1.91 ± 0.177	0.8299	ns
IL6	1.49 ± 0.059	1.7 ± 0.149	0.2282	ns
KLF10	1.09 ± 0.046	1.3 ± 0.108	0.1196	ns
KLF2	1.78 ± 0.062	1.53 ± 0.12	0.1113	ns
KLF3	1.01 ± 0.022	0.7 ± 0.032	<0.0001	****
KLF4	2.87 ± 0.199	1.58 ± 0.254	0.0016	**
LAMC2	1.17 ± 0.031	1.24 ± 0.142	0.6425	ns

MIB1	1.29 ± 0.033	1.16 ± 0.046	0.0432	*
NFKB1	1.02 ± 0.037	0.7 ± 0.059	0.0012	**
NFKBIA	0.89 ± 0.034	1.13 ± 0.102	0.0636	ns
NOTCH2	1.32 ± 0.113	2.62 ± 1.152	0.3123	ns
OCLN	0.94 ± 0.044	0.92 ± 0.095	0.8125	ns
PAPLN	1.5 ± 0.079	1 ± 0.083	0.0004	***
PECAM1	1.07 ± 0.02	0.91 ± 0.032	0.0017	**
PLA2G4A	1.4 ± 0.062	1.51 ± 0.173	0.5597	ns
PLXNA4	1.36 ± 0.207	0.58 ± 0.068	0.0008	***
PROCR	1.24 ± 0.033	0.92 ± 0.033	<0.0001	****
PTGIS	2.05 ± 0.239	1.84 ± 0.442	0.6864	ns
PTGS2	4.92 ± 0.255	6.57 ± 0.422	0.0083	**
S100A10	1.02 ± 0.017	0.82 ± 0.045	0.0051	**
SELE	1.12 ± 0.1	2.66 ± 0.298	0.0025	**
SMAD6	1.74 ± 0.108	1.52 ± 0.178	0.3217	ns
TGFB1	1.14 ± 0.03	1.14 ± 0.065	0.9822	ns
TJP1	0.93 ± 0.022	0.77 ± 0.048	0.0189	*
TNFRSF12A	1.19 ± 0.087	0.84 ± 0.171	0.1105	ns
TSPAN13	1.08 ± 0.033	0.84 ± 0.033	<0.0001	****
TUBB	1.04 ± 0.016	0.9 ± 0.036	0.0091	**
TXNIP	2.76 ± 0.397	0.98 ± 0.18	0.0002	***
VCAM1	1.92 ± 0.14	3.84 ± 0.482	0.009	**

***Supplementary Table 5: var* genotyping of patient-derived isolates after culturing by qPCR.** The primer sets detect multiple group A and A/B *var* domains and have been used to genotype the patient isolates at the time of isolation (original) (Storm et al, ref 26). For the UM and CM-derived isolates, genotyping was performed after a number of days in culture (DIC), as close to the DIC used for the co-culture experiments. The transcript unit (Tu) was calculated per primer set, relative to the transcripts of the endogenous housekeeping genes *seryl-tRNA synthetase* and *aldolase*, and for the combined (sum) CIDRα1 domains. A Tu value of 1 equals low abundance transcript and a value of 32 equals transcript levels as the endogenous housekeeping genes and values ≥32 are highlighted in bold in the dark grey cells. Tu values ≥5 correspond to moderate transcript levels and are highlighted in the light grey cells. Nd: not determined. Receptors for the PfEMP1 domains are listed with names in brackets as probable and left blank when unknown. For a detailed description of the domains see Mkumbaye et al [1].

**Domain**	**Group**	**Predicted receptor**	**UM1** Original	**UM1** 42 DIC	**UM2** Original	**UM2** 34	**UM3** Original	**UM3** 33 DIC	**UM3** 42	**UM4** Origina	**UM4** 26 DIC
CIDRα1.1	B/A	EPCR	8	1	5	1	2	3	1	5	1
CIDRα1.8a	B/A	EPCR	1	1	1	nd	1	1	1	nd	1
CIDRα1.8b	B/A	EPCR	1	1	1	1	1	1	1	nd	1
**Sum: CIDRα1.DC8**	B/A	EPCR	8	1	5	1	2	3	1	5	1
CIDRα1.4	A	EPCR	1	1	1	1	1	1	1	1	1
CIDRα1.5a	A	EPCR	1	1	1	1	1	nd	1	1	1
CIDRα1.5b	A	EPCR	4	3	2	1	1	1	1	3	1
CIDRα1.6a	A	EPCR	1	1	1	1	1	1	1	nd	1
CIDRα1.6b	A	EPCR	1	1	1	1	1	nd	1	nd	nd
CIDRα1.7	A	EPCR	4	1	2	1	5	1	1	1	1
**Sum: CIDRα1.A**	A	EPCR	8	4	4	1	5	1	1	4	1
**Sum:CIDRα1_all**	A & B/A	EPCR	16	5	8	1	7	3	1	10	1
DBLβ1/3-1	A	ICAM-1	2	1	1	1	1	10	1	5	3
DBLβ1/3-2	A	(ICAM-1)	9	1	3	1	12	17	1	**613**	**341**
DBLβ5	B	ICAM-1	1	1	3	2	1	5	1	2	5
DC5	A	PECAM-1	5	1	1	1	1	1	1	nd	3
CIDRα3.1/3.2	B/C	CD36	5	1	2	1	2	11	1	1	4
CIDRδ	A		2	1	1	1	3	4	2	nd	1
CIDRγ3.1	A		1	1	nd	1	1	1	1	nd	nd
DBLα1all	A		26	3	10	2	7	21	1	**296**	**222**
DBLα2/1.1/1.2/1.4/1.7	A		**36**	5	**33**	11	11	10	2	**109**	**73**
DBLα1.5/1.6/1.8	A	Non-EPCR	20	1	4	1	5	12	2	6	2
CIDRα1.2-K			1	1	nd	1	1	nd	1	nd	nd
CIDRα1.2-K+CIDRα1.3-K			1	1	1	1	1	1	1	nd	1

CM1 Original	CM1 28 DIC	CM1 29 DIC	CM2 Original	CM2 43 DIC	CM3 Original	CM3 51 DIC	CM4 Original	CM4 33 DIC
**53**	13	1	1	1	no data	1	**52**	2
6	nd	1	nd	1		1	1	1
**48**	**46**	1	nd	1		1	1	1
**107**	**59**	1	1	1		1	**52**	2
12	4	1	1	1		1	3	1
**33**	**53**	1	1	1		1	1	1
1	1	1	2	1		1	7	1
21	11	1	nd	1		1	7	1
1	3	nd	nd	1		1	1	nd
2	23	1	1	1		1	2	1
**68**	**94**	1	2	1		1	19	1
**175**	**154**	1	2	1		1	**72**	3
1	**55**	1	1	1		1	6	1
19	**815**	1	1	1		4	1	1
8	9	1	1	1		1	**33**	26
**66**	**86**	1	nd	1		1	2	1
5	27	1	5	1		1	7	7
8	**44**	1	nd	1		1	3	1
1	1	nd	nd	1		1	1	nd
**104**	**220**	1	**413**	4		1	**47**	9
**185**	**383**	4	12	1		5	**156**	14
**65**	**48**	1	**146**	3		1	**47**	3
1	nd	nd	nd	1		1	1	nd
3	2	1	nd	1		1	1	1

Supplementary Table 6: **Determination of prostaglandin endoperoxide synthase 2 (PTGS2) and prostacyclin production in HBMEC-IE co-cultures by ELISA.** Prostacyclin has a short half-life and therefore its hydrolysis product, 6-keto prostaglandin F1α (6-keto PGF1α), was detected in culture medium after co-culture with IE or RBC. PTGS2 was detected in HBMEC cell lysate after co-culture and calculated as ng PTGS2 per mg total lysate protein. The mean ± SD of 2 technical replicates is shown and per experiment the ratio of the enzyme concentrations for HBMEC-IE and HBMEC-RBC was calculated. 4 UM-derived isolates, 4 CM-derived isolates and SBP1-KO were co-cultured for 6 hours and for the CM4 isolate and SBP1-KO a co-culture of 20 hours was also performed (experiment 12 and 13). As control, HBMEC were incubated with 10 ng/ml TNF for 6 (experiment C2) and 20 hours (experiment C2 and C3). Thrombin is a fast-acting stimulator of prostacyclin production and the concentration of 6-keto PGF1α was detected in HBMEC after 15- and 30-minutes incubation with 5 nM thrombin (experiment C3). Statistical significance between HBMEC-IE and corresponding HBMEC-RBC and or control condition and medium was determined by unpaired t-test with Welch correction with * p-value < 0.05 and ** p-value < 0.01.

		[6ketoPGF1α] pg/ml		[PTGS2] ng/mg protein	
**Exp**	**Condition**	**Mean ± SD**	**IE/RBC**	**Mean ± SD**	**IE/RBC**
1	6h RBC	608 ± 43			
1	6h UM3-1	996 ± 136	1.64		
1	6h UM3-2	1046 ± 13 *	1.72		
1	6h CM2	1153 ± 271	1.90		
2	6h RBC	1621 ± 390			
2	6h UM1	952 ± 133	0.59		
2	6h CM3	1256 ± 162	0.77		
3	6h RBC	1679 ± 208			

3	6h CM1-1	1505 ± 73	0.90		
3	6h CM1-2	1611 ± 320	0.96		
4	6h RBC	437 ± 38			
4	6h UM2	803 ± 22 *	1.84		
5	6h RBC	664 ± 10		16.2 ± 0.3	
5	6h CM3	358 ± 100	0.54	19.1 ± 1.2	1.2
5	6h UM1	434 ± 56	0.65	10.8 ± 1.7	0.7
6	6h RBC	772 ± 182		18.0 ± 1.0	
6	6h CM4	694 ± 105	0.90	11.4 ± 0.6 *	0.6
7	6h RBC	1309 ± 265		19.0 ± 2.4	
7	6h UM3	1018 ± 79	0.78	10.0 ± 0.2	0.5
8	6h RBC	1194 ± 46		21.0 ± 0.6	
8	6h UM2	5720 ± 1378	4.79	17.4 ± 1.2	0.8
9	6h RBC	396 ± 19		19.4 ± 1.1	
9	6h CM1	695 ± 12 **	1.75	13.2 ± 1.3 *	0.7
10	6h RBC	1595 ± 114			
10	6h UM4	2013 ± 244	1.26		
11	6h RBC	4192 ± 1			
11	6h SBP1-KO-1	7213 ± 561	1.72		
12	6h RBC	1423 ± 109		13.7 ± 0.1	
12	6h CM4	1871 ± 98	1.32	12.5 ± 2.4	0.9
12	20h RBC	3754 ± 170		14.2 ± 1.5	
12	20h CM4	7382 ± 1233	1.97	9.0 ± 1.4	0.6
13	6h RBC	651 ± 11		15.3 ± 0.7	
13	6h SBP1-KO	480 ± 165	0.74	14.4 ± 2.1	0.9
13	20h RBC	2729 ± 113		13.2 ± 1.0	
13	20h SBP1-KO	8526 ± 1295	3.12	11.6 ± 3.1	0.9
C1	30 min medium	100 ± 0			
C1	15 min thrombin (5 nM)	1071 ± 104 *	10.74		
C1	30 min thrombin (5 nM)	826 ± 156	8.29		
C2	6h medium	264 ± 0			
C2	6h TNF (10 ng/ml)	909 ± 62 *	3.44		
C2	20h medium	685 ± 152			
C2	20h TNF (10 ng/ml)	2234 ± 1374	3.26		
C3	20h medium	178 ± 13		123.7 ± 14.9	
C3	20h TNF (10 ng/ml)	1200 ± 70 *	6.73	118.5 ± 19.4	1.0

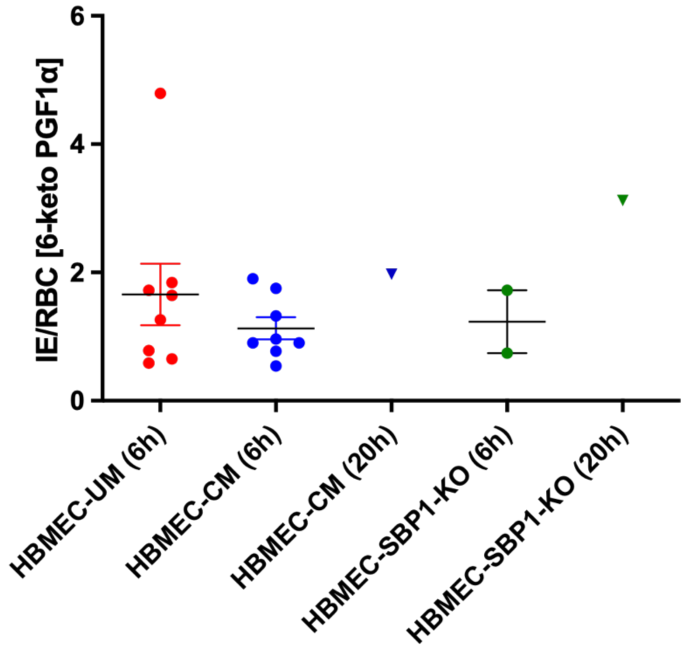

Supplementary Figure 3: **Ratio of 6-keto prostaglandin F1α concentrations in HBMEC-IE and HBMEC-RBC co-cultures.** 6-keto PGF1α was detected in culture medium after co-culture with IE or RBC and per experiment the ratio of the enzyme concentrations for HBMEC-IE and HBMEC-RBC was calculated (data in Supplementary Table 4). Plotted are mean ± SD of the ratios for the co-cultures with CM and UM-derived isolates and the SBP1-KO strain.

Supplementary Table 7: **Production of cytokines and chemokines in HBMEC-IE co-cultures.** A panel of 41 secreted cytokines and chemokines were measured in the co-culture medium of 6 hours co-culture of HBMEC-IE and HBMEC-RBC by Luminex. The results (pg/ml) are shown for 13 cytokines of which the concentration was higher than 5 pg/ml for 3 CM-derived isolates, 2 UM-derived isolates and 2 lab strains: IT4var14 and IT4var37. A positive control of HBMEC activated by 10 ng/ml TNF for 16 hours was included. The mean ± SD of two technical replicates is shown with significance between HBMEC-IE and HBMEC-RBC calculated by unpaired t-test with Welch correction, with * p-value < 0.05, ** p-value < 0.01 and *** p-value <0. 001.The ratio of the values of HBMEC-IE and HBEC-RBC was calculated per experiment. Note that the CM5 isolate was only included in this multiplex experiment and was not part of all the other experiments described.

	G-CSF	GM-CSF	Fractalkine	IFNg	GRO	PDGF-AA	IL-4	IL-6	IL-8	IP-10	MCP-1	RANTES	TNFa
**6h RBC**	120 ± 10	10 ± 1	165 ± 32	0.7 ± 0.5	567 ± 32	73 ± 5	3.5 ± 2.8	73 ± 60	956 ± 59	1089 ± 212	9162 ± 170	291 ± 22	10.1 ± 0.3
**6h CM1**	136 ± 10	13 ± 0	102 ± 0	8.0 ± 0.6 **	629 ± 22	85 ± 2	10 ± 4	99 ± 12	1371 ± 13	1209 ± 53	9837 ± 246	686 ± 42 *	10.2 ± 0.5
**IE/RBC**	1.1	1.2	0.6	10.8	1.1	1.2	2.9	1.4	1.4	1.1	1.1	2.4	1.0
**6h RBC**	369 ± 30	29 ± 2	341 ± 45	0.7 ± 0.0	2955 ± 108	75 ± 7	44 ± 0	136 ± 12	2293 ± 200	2686 ± 153	10754 ± 457	859 ± 5	15.5 ± 0.7
**6h CM4**	656 ± 14	49 ± 0.1	219 ± 22	7.4 ± 1.5	4572 ± 167	115 ± 1	55 ± 2	237 ± 3	3764 ± 93	3969 ± 28 *	13028 ± 965	980 ± 22	16.8 ± 1.4
**IE/RBC**	1.8	1.7	0.6	10.0	1.5	1.5	1.3	1.7	1.6	1.5	1.2	1.1	1.1
**6h RBC**	423 ± 42	32 ± 0	343 ± 10	1.2 ± 0.4	3167 ± 63	48 ± 3	41 ± 6	128 ± 29	4154 ± 94	1168 ± 22	12360 ± 560	392 ± 23	8.9 ± 0.1
**6h CM5**	715 ± 6	49 ± 3	161± 13 **	2.9 ± 0.6	3228 ± 197	75 ± 1	47 ± 0.1	170 ± 10	4436 ± 96	1353 ± 92	15720 ±1086	353 ± 343	9.5 ± 0.7
**IE/RBC**	1.7	1.5	0.5	2.5	1.0	1.6	1.1	1.3	1.1	1.2	1.3	0.9	1.1
**6h UM3**	805 ± 29	38 ± 2	137 ± 20 *	4.6 ± 0.0 *	2979 ± 143	90 ± 8 *	44 ± 1	155 ± 16	3876 ± 213	1834 ± 60 *	13676 ± 1404	396 ± 12	9.1 ± 0.4
**IE/RBC**	1.9	1.2	0.4	3.9	0.9	1.9	1.1	1.2	0.9	1.6	1.1	1.0	1.0
**6h RBC**	151 ± 5	19 ± 1	195 ± 24	1.1 ± 0.0	1308 ± 49	98 ± 7	21 ± 6	117 ± 10	1874 ± 68	1710 ± 30	10297 ± 58	709 ± 99	13.2 ± 0.3
**6h UM2**	213 ± 1	29 ± 1	107 ± 7	5.4 ± 0.2 *	994 ± 24	92 ± 2	18 ± 7	149 ± 7	2544 ± 77	1869 ± 70	10322 ± 386	666 ± 29	12.6 ± 0.1
**IE/RBC**	1.4	1.5	0.6	5.0	0.8	0.9	0.9	1.3	1.4	1.1	1.0	0.9	1.0
**6h RBC**	57 ± 2	9 ± 1	128 ± 7	1.3 ± 0.2	338 ± 3	56 ± 1	1.5 ± 0.0	18 ± 1	1228 ± 8	867 ± 72	7226 ± 366	591 ± 341	21.8 ± 1.5
**6h Var37**	61 ± 4	9 ± 1	105 ± 11	2.3 ± 0.2	264 ± 12	49 ± 9	5.5 ± 0.0 **	23 ± 4	1529 ± 145	836 ± 73	6240 ± 211	520 ± 40	12.0 ± 0.8
**IE/RBC**	1.1	1.0	0.8	1.8	0.8	0.9	3.6	1.3	1.2	1.0	0.9	0.9	0.6
**6h RBC**	212 ± 16	18 ± 0.3	120 ± 11	1.1 ± 0.5	1600 ± 90	50 ± 3	24 ± 0	105 ± 5	1849 ± 38	1262 ± 110	12755 ± 1427	495 ± 7	9.8 ± 0.0
**6hVar14**	328 ± 22	26 ± 3	71 ± 13	1.6 ± 0.2	1830 ± 235	48 ± 9	29 ± 7	114 ± 18	1972 ± 103	1099 ± 88	12509 ± 1434	460 ± 138	9.9 ± 1.2
**IE/RBC**	1.5	1.4	0.6	1.5	1.1	1.0	1.2	1.1	1.1	0.9	1.0	0.9	1.0
**medium overnight**	36 ± 0	1 ± 0.3	23 ± 17	0.4 ± 0	266 ±18	94 ± 16	5.5 ± 0.0	20 ± 2	158 ± 3	12 ± 1	1056 ± 43	1.3 ± 0.4	0.2 ± 0.2
**TNF overnight**	nd	1385 ± 17 **	2227 ± 25 ***	1.6 ± 0.2	49974 ± 1763 *	138 ± 18	170 ± 6 *	1308 ± 45 *	20734 ± 3283	10465 ± 155 **	13783 ± 1365 *	925 ± 9 **	11068 ± 486 ***
**TNF/medium**		1372	190	4	188	1.5	31	65	131	864	13	718	52410

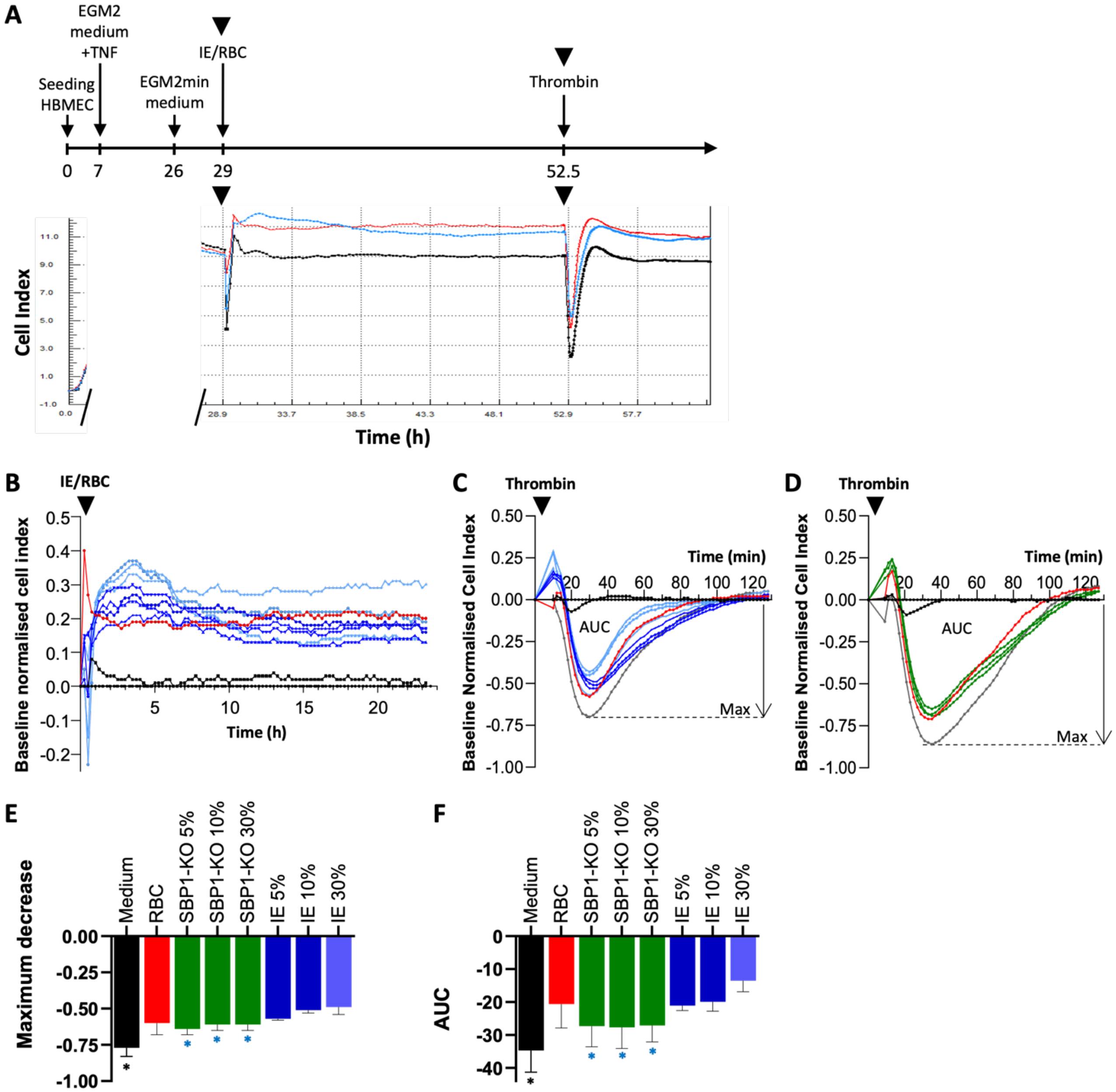

Supplementary Figure 4: **The effect of the patient-derived isolates and SBP1-KO on HBMEC barrier function determined by trans endothelial electrical resistance.** (A) Experimental timeline (not to scale) and a representative cell index trace for IE and thrombin induced changes in HBMEC barrier function. HBMEC were seeded in the E-plate and after approximately 7 hrs the cells were stimulated with 10 ng/ml TNF. The next day, medium was replaced with EGM2min medium and after 2-3 hours, IE at 30% parasitaemia (light blue) or RBC (red), both at 1% HCT, were added. The black line represents medium. After approximately 24 hrs, 5 nM thrombin was added from a concentrated stock solution and cell index was monitored for an additional 10 hours. (B) Cell index trace for the effect of different patient isolates and RBC on HBMEC barrier function of a representative experiment. Cell index was normalised at the time point immediately prior to addition of RBC and IE, indicated by the black triangle, and medium only (black line) was set as baseline. This baseline normalised cell index (BNCI) is shown for 2 UM and 2 CM-derived isolates at 10% parasitaemia (dark blue), 1 UM and 2 CM-derived isolates at 30% parasitaemia (light blue), RBC (red) or replicate medium (black). (C) Thrombin induced decrease in barrier function after 24-hour exposure to patient isolates or RBC. Cell index was normalised at the time point immediately prior to addition of 5 nM thrombin, indicated by the black triangle, and medium without thrombin (black line) was set as baseline. BNCI is shown for medium (dark grey) with the maximum response indicated (max), RBC (red), 1 UM and 2 CM-derived isolates at 30% parasitaemia (light blue) and at 10% parasitaemia (dark blue). (D) As in panel C, but with SBP1-KO at 30% and 10% (green). (E) Maximum decrease of BNCI by thrombin was determined for all co-culture

conditions and mean ± SD are depicted for: control medium (n = 4), RBC (n = 4), SBP1-KO at 5% (n = 3), 10% (n = 3) and 30% parasitaemia (n = 3) and combined patient isolates (IE) at 5% (n = 2), 10% (n = 5) and 30% parasitaemia (n = 5). (F) Area under the curve (AUC) after thrombin induced changes in BNCI was calculated and mean ± SD are depicted for the conditions listed in E. Statistical significance between the various conditions compared to RBC was calculated by unpaired t-test with Welch correction, * p-value < 0.05 (black asterisk). Significance between 30% IE and SBP1-KO was also calculated (blue asterisk).

Supplementary Methods

var Genotyping of Patient-Derived Isolates after Culturing

Genotyping of the isolates after culturing was performed by qPCR using selected primers from a *var* gene primer panel as described by Storm et al [2], with details of the primer design and description of the var domains described by Mkumbaye et al [1]. RNA was isolated from TRIZOL-stored ring stage IE, DNase treated (TURBO™ DNase, Ambion), cDNA synthesised (Tetro cDNA Synthesis Kit, Bioline) and qPCR carried out with SYBR Green PCR Master Mix (QuantiTect, Qiagen). The endogenous housekeeping genes *seryl-tRNA synthetase* and *aldolase* were used to determine the relative levels of *var* transcripts using the formulae ΔCt *var*-primer = Ct *var* primer minus Ct average of endogenous primers. The transcript unit (Tu) was then calculated as $Tu = 2^{(5-\Delta Ct\ var\text{-}primer)}$. Any low abundance transcripts with a ΔCt *var*-primer >5 was assigned a value of 5, thus subsequently a Tu value of 1. A Tu value of 32 equates to equal transcript levels as the endogenous control genes [1]. The Tu of the patient isolates at the time of isolation from peripheral blood was determined previously [2].

Preparation of HBMEC-IE Co-culture Samples for Phenotypic Analysis

After 6 or 20 hour co-culture with patient isolates, SBP1-KO, RBC or additional controls, co-culture medium was collected, centrifuged for 5 min at 900 g to remove IE, RBC or cellular debris and the supernatant frozen at -80 °C. For cell lysates, HBMEC were washed twice with warm EGM2 medium without supplements and lysed in 50 µl lysis buffer (RIPA buffer (Sigma, R0278) with HALT™ protease inhibitor cocktail (Thermo Scientific, 78429) for 10 minutes on ice. The lysed cells were transferred from the well into a tube, placed on ice for an additional 25 minutes, centrifuged for 5 min at 13,000 g at 4 °C and the supernatant frozen at -80 °C.

Determination of the Concentrations of Prostacyclin and Prostaglandin Endoperoxide Synthase 2

Prostacyclin has a short half-life and therefore its hydrolysis product, 6-keto prostaglandin F1α (6-keto PGF1α), was detected in co-culture medium by competitive ELISA according to the manufacturer's instructions (Cayman Chemicals, 515211). The four UM and four CM-derived isolates and SBP1-KO were co-cultured for 6 hours with their respective RBC as control. The CM4 isolate and SBP1-KO were also co-cultured for 20 hours. As additional controls, HBMEC were incubated with 10 ng/ml TNF for 6 and 20

hours and with 5 nM thrombin for 15 and 30 minutes. Co-culture medium of two independent 6 hour co-culture experiments with the patient isolates, except UM4 and CM2, and SBP1-KO were used, and all samples were measured in duplicate using 2 ELISA plates on the same day. All samples were diluted 2 times, but the 20 hour TNF stimulation sample was diluted 5 times. The concentration of 6-keto PGF1α was calculated using the provided standards.

Prostaglandin endoperoxide synthase 2 (PTGS2) was detected in HBMEC cell lysate by ELISA according to the manufacturer's instructions (Reddot, RD-PTGS2-Hu). Lysates were diluted 5 times and for duplicate samples the concentration of PTGS2 was calculated using the provided standards. On the same day, total protein concentration of the lysates was determined with the BCA kit (Biorad, 5000002) with BSA as standard and ng PTGS2 per mg total protein calculated.

Detection of Cytokines and Chemokines

A panel of 41 secreted cytokines and chemokines was measured in the co-culture medium of 6 hours co-culture of HBMEC-IE, HBMEC-RBC and controls using the human cytokine/chemokine 41 plex Immunology Multiplex Assay according to the manufacturer's instructions (Merck, HCYTMAG-60K-PX41). In addition to three CM and two UM-derived isolates, two lab strains, IT4var14 and IT4var37, and a positive control of HBMEC activated by 10 ng/ml TNF for 16 hours were included. The panel included: sCD40L, Eotaxin, FLT-3L, Fractalkine, G-CSF, GM-CSF, GROα, IFNα2, IFNγ, IL-1α, IL-1β, IL-1RA, IL-2, IL-3, IL-4, IL-5, IL-6, IL-7, IL-8, IL-9, IL-10, IL-12 (p40), IL-12 (p70), IL-13, IL-15, IL-17A, IL-17E/IL-25, IL-17F, IL-18, IL-22, IL-27, IP-10, MCP-1, MCP-3, M-CSF, MDC, MIG, MIP-1α, MIP-1β, PDGF-AA, PDGF-AB/BB, RANTES, TGFα, TNFα, TNFβ, VEGF-A.

Measuring HBMEC Barrier Integrity

Barrier function was measured by real time Trans Endothelial Electrical Resistance (TEER) analysis with the xCELLigence® RTCA S16 system (ACEA Biosciences). HBMEC cells were seeded at 50,000 cells/cm^2 ($1x10^4$ cells) in Attachment Factor coated E-Plates 16 PET (ACEA Biosciences) in EGM2 medium and the arbitrary cell index (CI) was recorded. After approximately 7 hrs the medium was replaced with EGM2 medium with 10 ng/ml TNF and the next day, medium was replaced with EGM2min medium. IE suspensions at 30%, 10% or 5% parasitaemia were prepared at 1% HCT in EGM2min medium and the medium in the E-plate replaced with 100 µl suspension, equivalent to 3x106, 1x106 and 0.5x106 IE, respectively. RBC at 1% HCT and medium only were used as control.

After approximately 24 hrs, 5 nM thrombin was added from a concentrated stock solution and cell index was monitored for an additional 10 hours, starting at 2 minute intervals. For analysis, the cell index was

normalised either at the time point immediately prior to addition of IE or thrombin (normalised cell index) and medium only was set as baseline (baseline normalised cell index, BNCI). To determine the effect of thrombin, the maximum decrease in BNCI and the area under the curve after recovery to baseline was calculated.

Supplementary References

1 Mkumbaye, S. I. *et al.* The Severity of Plasmodium falciparum Infection Is Associated with Transcript Levels of var Genes Encoding Endothelial Protein C Receptor-Binding P. falciparum Erythrocyte Membrane Protein 1. *Infect Immun* **85**, doi:10.1128/IAI.00841-16 (2017).

2 Storm, J. *et al.* Cerebral malaria is associated with differential cytoadherence to brain endothelial cells. *EMBO Mol Med* **11**, doi:10.15252/emmm.201809164 (2019).
